# High-resolution profiling of linear B-cell epitopes from mucin-associated surface proteins (MASPs) of *Trypanosoma cruzi* during human infections

**DOI:** 10.1371/journal.pntd.0005986

**Published:** 2017-09-29

**Authors:** Ignacio M. Durante, Pablo E. La Spina, Santiago J. Carmona, Fernán Agüero, Carlos A. Buscaglia

**Affiliations:** Instituto de Investigaciones Biotecnológicas-Instituto Tecnológico de Chascomús (IIB-INTECh), Universidad Nacional de San Martín (UNSAM) and Consejo Nacional de Investigaciones Científicas y Técnicas (CONICET); Buenos Aires, Argentina; Instituto de Ciências Biológicas, Universidade Federal de Minas Gerais, BRAZIL

## Abstract

**Background:**

The *Trypanosoma cruzi* genome bears a huge family of genes and pseudogenes coding for Mucin-Associated Surface Proteins (MASPs). MASP molecules display a ‘mosaic’ structure, with highly conserved flanking regions and a strikingly variable central and mature domain made up of different combinations of a large repertoire of short sequence motifs. MASP molecules are highly expressed in mammal-dwelling stages of *T*. *cruzi* and may be involved in parasite-host interactions and/or in diverting the immune response.

**Methods/Principle findings:**

High-density microarrays composed of fully overlapped 15mer peptides spanning the entire sequences of 232 non-redundant MASPs (~25% of the total MASP content) were screened with chronic Chagasic sera. This strategy led to the identification of 86 antigenic motifs, each one likely representing a single linear B-cell epitope, which were mapped to 69 different MASPs. These motifs could be further grouped into 31 clusters of structurally- and likely antigenically-related sequences, and fully characterized. In contrast to previous reports, we show that MASP antigenic motifs are restricted to the central and mature region of MASP polypeptides, consistent with their intracellular processing. The antigenicity of these motifs displayed significant positive correlation with their genome dosage and their relative position within the MASP polypeptide. In addition, we verified the biased genetic co-occurrence of certain antigenic motifs within MASP polypeptides, compatible with proposed intra-family recombination events underlying the evolution of their coding genes. Sequences spanning 7 MASP antigenic motifs were further evaluated using distinct synthesis/display approaches and a large panel of serum samples. Overall, the serological recognition of MASP antigenic motifs exhibited a remarkable non normal distribution among the *T*. *cruzi* seropositive population, thus reducing their applicability in conventional serodiagnosis. As previously observed in *in vitro* and animal infection models, immune signatures supported the concurrent expression of several MASPs during human infection.

**Conclusions/Significance:**

In spite of their conspicuous expression and potential roles in parasite biology, this study constitutes the first unbiased, high-resolution profiling of linear B-cell epitopes from *T*. *cruzi* MASPs during human infection.

## Author summary

Trypanosomatids belong to an ancient eukaryotic lineage that cause devastating diseases in humans and livestock. The elucidation of the genomes of *Trypanosoma cruzi*, *Trypanosoma brucei* and *Leishmania major* established a landmark in the study of these relevant protozoa. In particular, the genome of *T*. *cruzi*, the agent of Chagas disease, evidenced a marked expansion and diversification of gene families likely involved in parasite-host interplay. MASPs (for mucin-associated surface proteins) define the second largest *T*. *cruzi* gene family, with ~1400 genes (and pseudogenes) coding for surface glycoproteins involved in parasite infectivity and immune evasion. MASP deduced products bear highly conserved sorting signals on their N- and C-terminal regions that direct and anchor them to the parasite plasma membrane. Conversely, the central and mature domain, the only one displayed on the parasite surface is strikingly variable and exhibit a ‘mosaic-like’ structure made up of different combinations of a large repertoire of short sequence motifs. Few studies have analyzed MASP antigenicity so far, and they were carried out using either low throughput approaches or animal infection models. By means of exquisite peptide micro-arrays technology followed by conventional serological methods we herein present the first non-biased, high-throughput profiling of linear B-cell epitopes from a representative pool (n = 232) of *T*. *cruzi* MASPs during human infection. Our findings are discussed in terms of MASP evolution, expression profile and applicability in Chagas disease serodiagnosis.

## Introduction

Trypanosomatids constitute an early branch of the eukaryotic lineage, which includes several protozoan parasites transmitted by hematophagous insect vectors that cause devastating diseases in humans as well as countless infections in livestock and wild vertebrates, primarily in developing countries [1,2]. The simultaneous release of the ‘tritryps’ genomes, i.e. the complete DNA sequences of *Trypanosoma cruzi* [3], *Trypanosoma brucei* [4] and *Leishmania major* [5] in 2005 established a landmark in the study of this important group of organisms. In comparison, the genome of *T*. *cruzi*, the etiological agent of Chagas disease, revealed a remarkable expansion and diversification of certain gene families likely involved in its interaction with the mammal and/or vector hosts [3]. These included the previously characterized gp85/*trans*-Sialidase (TS)-like molecules [6,7], mucins [8,9], dispersed gene family 1 (DGF-1) [10], and gp63 metalloproteases [11,12]. The *T*. *cruzi* genome also allowed for the identification of a group of ~1,400 highly polymorphic genes (including ~400 pseudogenes), coding for a novel family of putative surface glycoproteins [3]. Some of them were located immediately downstream of *TcMUC* genes, which code for the polypeptide scaffolds of mucins from bloodstream trypomastigotes [13,14], and were therefore termed MASPs (for mucin-associated surface proteins) [3,15]. Due to the particular type of gene expression regulation in trypanosomatids, in which nearly all protein-coding genes are arrayed in long polycistronic transcription units [16], this genomic disposition hinted at a possible co-expression of *MASP* and *TcMUC* genes on the surface of trypomastigotes. Subsequent transcriptomic and proteomic studies supported this assumption and unveiled distinctive *MASP* expression features such as concurrent expression of multiple ‘alleles’ in a single parasite population, and variations in the subset of members preferentially transcribed among trypomastigotes from a single population [15,17–22].

Deduced MASP products are flanked by conserved motifs coding for an *N*-terminal signal peptide (SP) and a *C-*terminal glycosylphosphatidyl inositol (GPI) anchoring signal. These sequences ensure the proper traffic through the secretory pathway and posterior tethering of MASP polypeptides to the outer leaflet of the *T*. *cruzi* membrane [15]. In addition, MASP products were also found associated to secreted plasma membrane-derived micro-vesicles (MVs) [23–25]. Intriguingly, and even though trypanosomatids have evolved fine-tuned transport mechanisms for efficient processing and surface display of large amounts of GPI-anchored molecules [26,27], ‘immature’ MASPs bearing non-cleaved sorting signals were recently found inside MVs [24,25]. Within the central and ‘mature’ region, i.e. the only region displayed on the parasite surface upon canonical intra-cellular processing, MASPs show great variability both in size and amino acid sequence [3,15]. Most of them display repetitive motifs and are predicted to undergo multiple post-translational modifications, including Ser/Thr-phosphorylation and glycosylation [15]. Proteomic datasets have indeed revealed the presence of several MASP-derived glycopeptides, some of which bear terminal sialic acid residues [19,21,23,28].

A distinctive feature of MASP products is that they present a ‘mosaic-like’ structure, made up of different combinations of a large repertoire of short sequence motifs [3,15]. Such an arrangement probably emerged as a result of multiple rounds of gene duplication followed by diversification and intra-family recombinational events. In this context, it is worth noting that *MASP* genomic clusters are also enriched in retro-elements and members of the *T*. *cruzi* retrotransposon hot spot (RHS) family [3,29], which may have fostered *MASP* recombination. A similar kind of evolutionary pathway has been proposed for gp85/TS-like molecules [30] as well as for certain multi-gene families of different pathogens, such as *T*. *brucei* VSGs [31], the *var* genes of the human malaria parasite *Plasmodium falciparum* [32] and the α-like proteins of *Streptococcus agalactiae* [33]. This trait is supposed to have evolved as a means to evade the mammalian host immune system and/or to expand the range of pathogens’ interactions with the host, thereby increasing their persistence and chronicity [34].

In spite of their genomic predominance [15], recurring identification in proteomic surveys [19–21,23,28,35] and potential roles in trypomastigote protection [36] and/or virulence [37–39] the antigenicity of MASP family members has only recently been analyzed. After assessing the expression profile of *MASP* genes in cell-derived trypomastigotes, the group of Dr. Bartholomeu analyzed the antigenicity of a number of selected MASP-derived peptides [18]. Although this work served to demonstrate that different MASP members constitute parasite antigens that are recognized by IgG and IgM antibodies, the selection of peptides was biased by the use of a bioinformatics prediction algorithm and was carried out in a mice model of acute *T*. *cruzi* infection [18]. An unbiased, high-content study aimed at characterizing the antigenic profile of the MASP family in *T*. *cruzi*-infected humans is still lacking. Recently, in the context of a project aimed at identifying novel *T*. *cruzi* linear B-cell epitopes, we produced high-density peptide microarrays (henceforth Chagas-chip), which were screened with serum samples from chronic Chagasic patients [40]. Using completely overlapped 15mer peptides, the Chagas-chip spanned the entire sequence of 232 non-redundant MASP deduced products, accounting for ~25% of the total MASP content of the CL Brener genome reference clone [40]. Here, we present a detailed antigenic analysis of Chagas-chip-sampled MASPs, which allowed for the identification, mapping at maximal resolution, and overall characterization of their most relevant linear B-cell epitopes, as observed in humans. The serodiagnostic performance of peptide motifs showing top-ranking reactivity was further evaluated using distinct synthesis/display approaches and a large panel of human serum samples.

## Materials and methods

### MASP sampling

Design and synthesis of the Chagas-chip, and the different protein groups included in the array have been described [40]. Group3 of this array included a curated list of 232 non-redundant MASPs, which could be split into 2 major subsets according to our inclusion criteria. Subset 1 or ‘MEMEs’ comprised 136 MASPs randomly selected from the *T*. *cruzi* CL Brener genome draft. In the absence of structural features allowing definition of coherent and robust intra-family groups, and aiming to maximize the coverage of MASP variability, one representative member of each of the 136 MEMEs (Multiple Expectation Maximization for Motif Elicitation) groups defined in [3] was chosen. Subset 2 comprised 96 MASPs with previous evidence of mRNA and/or protein expression. This subset included 14 MASPs identified in a trypomastigote cDNA library [15]; 8 MASPs bearing a peptide displayed on the surface of trypomastigotes as assessed by a specific antibody [15]; and 74 MASPs bearing peptides identified on different proteomic surveys [17,23,28]. Overall, and except for 5 sequences that emerged from proteomic datasets of *T*. *cruzi* insect vector-dwelling stages [17], this subset was strongly biased towards trypomastigote-expressed sequences and was hence termed ‘Trypomastigote’. The complete list of analyzed MASPs is included in S1 Table. The entire amino acid sequences of these MASP molecules, as annotated in the TritrypsDB Database resource [41], were spanned with fully overlapped 15mer peptides (1 residue shift), and synthesized *in situ* in different positions in the array.

### Screening of Chagas-chips

Assay and analysis of Chagas-Chips was described thoroughly previously [40]. Briefly, the Chagas-chip was firstly assayed with the negative sample (pooled IgG purified from 5 healthy subjects that yielded negative results for *T*. *cruzi* conventional serological tests) and then with the positive sample, composed of pooled IgGs purified from 5 individuals coursing the chronic phase of Chagas disease, with no cardiac involvement or other Chagas disease-associated pathology [40]. Two data sets were therefore obtained, one corresponding to the readout from healthy individuals (negative control) and one corresponding to the accumulated signal of the negative plus positive samples. Reactivity of positive samples was then calculated by subtraction. Four independent experiments differing in the composition of the positive sample were carried out, each one in duplicate. A cutoff of 3 arbitrary units of fluorescence (range 2.6 to 3.4 after normalization and smoothing) was established from whole-chip analyses, as it yielded optimal sensitivity and specificity towards included controls [40].

### Identification and characterization of MASP antigenic motifs

After data treatment and analysis, we identified consecutive stretches of reactive MASP-derived peptides defining antigenic peaks (S1 Fig and [40]). Peptides showing the highest microarray average reactivity within each antigenic peak (henceforth MRPs, for most reactive peptides) were defined as the antigenic motif cores and used to calculate the overall reactivity for each positive MASP as the sum of their individual MRP scores (S1 Fig). MRPs were aligned using the ClustalW algorithm to obtain a preliminary phylogenetic tree. An identity matrix was obtained and peptide clusters with ≥ 50% internal identity were identified. To visualize the clusters, a simplified cladogram derived from the online tool *Phylogeny*.*fr* [42] is shown. For those clusters bearing ≥ 3 sequences, antigenic motifs were refined by carrying out ClustalW alignments of MRPs within each cluster, and graphically depicted as sequence logos [43] constructed by the online server weblogo.berkeley.edu [44], and edited for publication. Genomic analysis of antigenic motifs was performed by strict motif-oriented homology searches in the *T*. *cruzi* CL Brener, Dm28c and Sylvio X-10 isolates genome sequences annotated in the Jan-2017 tritrypdb.org repository [41]. To assess the position of MRPs independently of the variable length of any given MASP, a relative position (r.p.) index (ranging from 0 to 1) was calculated as follows: r.p. = *C*-terminal position of MRP / total length of the corresponding MASP. In each case, the annotated translation initiation Meth residue [41] was scored as position 1. To normalize the reactivity score of MRPs, a relative score (r.s.) index (ranging from 0 to 1) was computed as follows: r.s. = score of MRP / score of most reactive MRP (64.57, peptide sequence QVAGIKTTTATTGDS). Positional evaluation of MRPs within MASPs was done by plotting r.s. *vs*. r.p. values for each MRP. Correlation was assessed using the Pearson’s R correlation coefficient as implemented in GraphPad Prism software.

### Recombinant protein generation and purification

For serological validation purposes, MASP-derived antigenic motifs identified in the Chagas-chip were firstly prioritized as indicated in text. Different motifs were then selected and produced in bacteria as Glutathione *S*-transferase (GST)-fusion proteins. Briefly, gene amplifications were done by PCR using 1–10 ng of phenol-chloroform purified *T*. *cruzi* CL Brener genomic DNA as template and Taq DNA Polymerase High Fidelity (Stratagene) [45]. Due to the complexity of the *MASP* gene family [3,15], several oligonucleotide primer combinations were tested *in silico* in order to maximize amplification specificity. In most cases, they were designed to align to DNA sequences flanking the target antigenic motif whereas in others, such as in motif 1, they had to be synthetized as partially complementary sequences spanning solely the motif. Sequence and features of oligonucleotides and of their resulting amplicons are compiled in S2 and S3 Tables, respectively. Amplicons were purified, cloned into pGEM-T easy vector (Promega) and used to transform TOP10F cells (Invitrogen). They were then sub-cloned into the XhoI and NotI restriction sites of a tailored version of the pGEX-1*λ*T (GE Healthcare) vector in which the sequence of its multiple cloning site had been previously modified with partially complementary oligonucleotides PGEX1 and PGEX2 (S2 Table). Cloning was checked by restriction mapping analysis and DNA sequencing. *Escherichia coli* strain BL21-CodonPlus (Stratagene) were transformed with each construct and induced for 3 h at 28°C with 0.1 mM isopropyl-β-d-thiogalactopyranoside (Fermentas). After bacterial lysis in 50 mM Tris pH 7.5 150 mM NaCl 0.05% NP-40, supernatants were purified by glutathione-Sepharose chromatography (GE Healthcare) and dialyzed against phosphate-buffer saline (PBS). GST-fusion molecules were quantified using the Bradford reagent (Pierce), according to manufacturer’s indications, and purity was assessed by Coomassie brilliant blue-stained SDS-PAGE.

### Synthetic peptides

Standard FMOC-synthesized peptides (>90% purity) corresponding to different MASP antigenic motifs were purchased from Genescript (NJ, USA). Sequences and features of these peptides are indicated in S3 Table. Peptides were resuspended in PBS and, when indicated, coupled to maleimide-activated Bovine Serum Albumin (BSA, Thermo) through an additional *C*-terminal Cys residue as described [46].

### ELISA (Enzyme-Linked Immunosorbent Assay) and competitive ELISA

Individual MASP sequences (expressed either as GST-fusion proteins or BSA-coupled peptides) were dissolved in carbonate buffer (pH 9.6) at 10 μg/ml, and 100 μl of this solution was used to coat flat-bottomed 96-well Nunc-Immuno plates (Nunc, Roskilde, Denmark). Following an overnight incubation at 4°C, plates were washed 3 times with PBS containing 0.05% Tween 20 (PBS/T), blocked for 1 h with 4% skim milk in PBS/T at 37°C, and processed for ELISA as described [47]. Each serum sample was diluted 1:1,000 in 4% skim milk PBS/T buffer and assayed in triplicate. A peptide spanning the antigenic region (residues 30 to 50) from the extensively characterized *T*. *cruzi* CL Brener TSSA (Trypomastigote Small Surface Antigen) molecule [47–50], and a scrambled version of this peptide were used as internal positive and negative controls, respectively (S3 Table). Sequences for both peptides has been described [47]; and they were coupled to BSA and assayed as described above. GST was used as internal negative control for GST-fusion proteins. To assess inter-assay variability, cutoff and sample values were relativized to a positive control (a serum sample from a chronic Chagasic patient yielding 0.8–1.4 absorbance units towards TSSA) included in each assay [47]. Results were considered positive if read mean absorbance was above the cutoff (i.e. mean absorbance + 3 SD) calculated for negative sera. For competitive ELISA tests, serum samples were diluted up to 10 μl in PBS containing different amounts (0, 0.1, 1 and 10 μg) of the indicated synthetic peptide and incubated for 30 min at room temperature before being diluted to 1:1,000 in 4% skim milk PBS/T and added to GST-fusion protein-coated ELISA plates. Absorbance at 450 nm in the control wells in which the serum samples were incubated with PBS without peptide was taken as 100% reactivity [47].

### Human sera

A panel of serum samples from 58 chronically infected patients was obtained from the Instituto Nacional de Parasitología “Dr. Mario Fatala Chabén” (Buenos Aires, Argentina). These serum samples yielded positive results when analyzed for *T*. *cruzi*-specific antibodies with the following commercially available kits: ELISA using total parasite homogenate (Wiener lab, Argentina) and indirect hemagglutination (HAI, Polychaco, Buenos Aires, Argentina) and have been described in [50]. The negative panel was composed of 30 samples from healthy individuals that gave negative results in the aforementioned tests, and were obtained from the blood bank Fundación Hemocentro Buenos Aires (Buenos Aires, Argentina).

### Ethics statement

The Institutional Review Board of UNSAM has evaluated the current project and considered that it complies with the Basic HHS Policy for Protection of Human Research Subjects requirements to be included in the ‘exemption 4’, because it involved the use of de-coded and de-identified human serum samples obtained from sera repositories where they were preserved for diagnosis studies purposes.

## Results

### High-throughput discovery of antigenic MASPs

**Fig 1:**
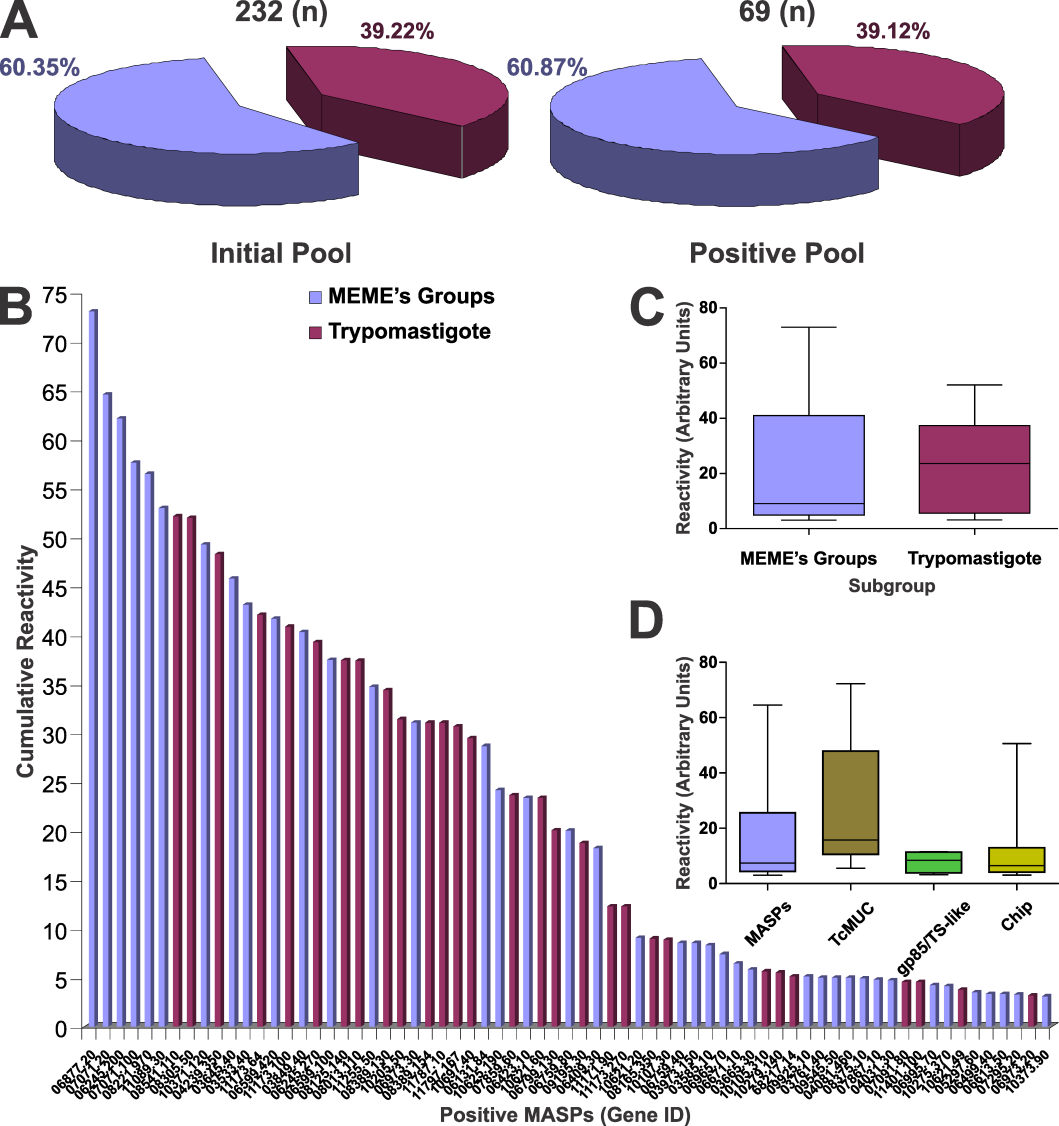
High-throughput discovery of antigenic MASPs. **A)** Pie charts showing the total MASP content and representation of subgroups (MEMEs, Trypomastigote) included in the initial pool (left) and the resulting pool after Chagas-chip serological evaluation (right). **B)** Column chart showing the cumulative reactivity values for each of the 69 positive MASPs. The original affiliation of each MASP (either to the MEMEs or Trypomastigote subgroup) is indicated with a color code as in panel A. **C)** Box-and-whiskers chart comparing the Chagas-chip average reactivity for MEMEs and Trypomastigote subgroups. **D)** Box-and-whiskers chart comparing the Chagas-chip average reactivity for positive MASPs, TcMUC, gp85/TS-like molecules, and the overall positive proteins in the Chagas-chip (Chip).

Upon 4 independent serologic evaluations of the Chagas-chip and downstream data analysis, we identified 790 MASP-derived peptides defining 86 antigenic peaks, as recognized by antibodies present in sera from human subjects carrying asymptomatic infections with *T*. *cruzi*. These peptides were mapped to 69 out of the 232 (∼30%) sampled MASPs (Fig 1A and [40]). Antigenic peaks encompassed a stretch of adjacent peptides in a protein sequence with above-the-threshold reactivity for at least 1 assay, and usually define a single B-cell linear epitope. In exceptional cases, as demonstrated for the TSSA antigen, particularly broad antigenic peaks may contain a few partially overlapped epitopes [47]. The pool of MASPs analyzed in the Chagas-chip included sequences randomly selected from the *T*. *cruzi* deduced proteome (the ‘MEMEs’ subgroup) and a subset of sequences with previous evidence of being expressed in parasites, mostly in trypomastigote forms (termed ‘Trypomastigote’). Even though MASPs from the latter subset were in principle more prone to elicit immune responses in *T*. *cruzi*-infected individuals, the relative representation of either subset in the positive pool remained relatively unaltered with respect to that of the original pool (Fig 1A). Solely upon assessing the cumulative reactivity of the 69 positive MASPs (calculated as the sum of their individual antigenic peaks scores, S1 Fig), a certain though not significant trend towards higher reactivity for ‘Trypomastigote’-comprised members was found (Fig 1B and 1C). Overall, positive MASPs showed similar patterns of reactivity (in terms of median and dispersion of values) as compared to other *T*. *cruzi* complex protein families evaluated in the Chagas-chip such as TcMUC and gp85/TS-like molecules (Fig 1D). These parameters were not significantly different from those assessed from the overall reactive proteins included in the Chagas-chip (Fig 1D).

### Identification and characterization of MASP antigenic motifs

**Fig 2:**
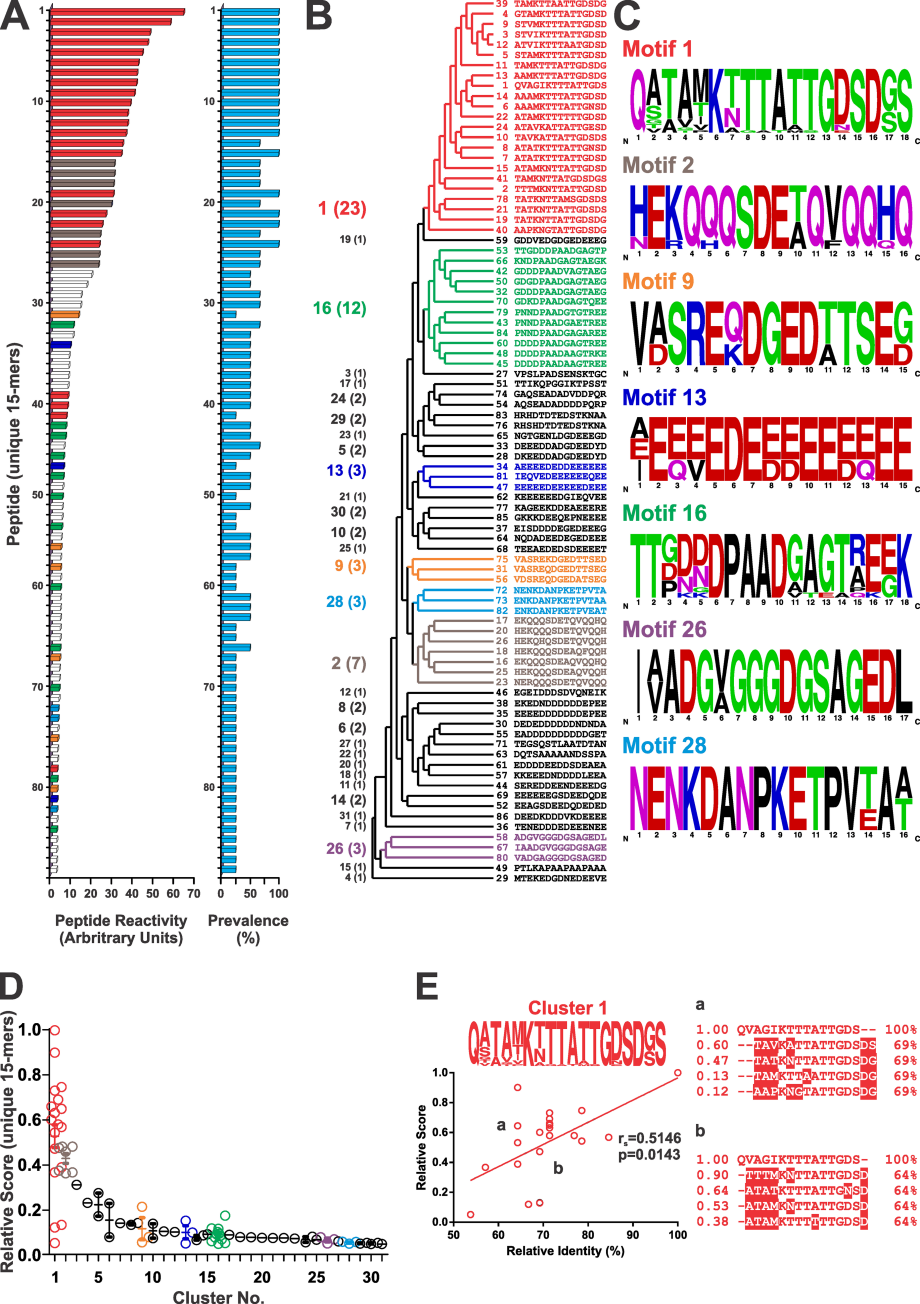
Reactivity and clustering analysis of positive MASP peptides. **A)** Column chart showing Chagas-chip mean reactivity values for the most reactive peptide (MRP) within each positive peak (n = 86). The Chagas-chip prevalence calculated for each MRP is also indicated in the column chart of the right. **B)** Simplified cladogram showing the clustering of MRPs. Clusters numbers and *n* values are indicated to the left. **C)** Consensus antigenic motifs corresponding to clusters with *n* ≥ 3 (color-coded as in panel A) are shown as *WebLogo* graphics derived from the alignment of constituent sequences. Clusters with *n* < 3 (in white in panel A) are shown in black. **D)** Dispersion chart showing relative reactivity values for every MRP within each cluster. **E)** Analysis of most relevant antigenic positions from cluster 1. Relative antigenicity of peptides is plotted as function of sequence identity (%) relative to the most reactive peptide within this group. ClustalW alignments between two subgroups (a,b) of peptides with similar % identity displaying great dispersion in their reactivity. Divergent positions in relation to the first sequence are boxed.

Given the peculiar ‘mosaic-like’ structure of the MASP family of proteins [3], we reasoned it would be more informative to proceed with our antigenic characterization at the antigenic motif rather than whole polypeptide level. To that end, peptides showing the highest microarray average reactivity (MRPs) were identified within each antigenic peak. As shown in Fig 2A, MRPs showed great dispersion on their reactivity values, with ~50% of them yielding signals slightly above the established cutoff. They also showed variations in their Chagas-chip prevalence, i.e., fraction of positive results out of 4 independent screenings using distinct chronic Chagasic sera (Fig 2A). Upon conducting a similarity-based analysis, the 86 MASP-derived MRPs were classified into 31 clusters, considering a cutoff of 50% of sequence identity within each cluster (Fig 2B). Composition of the clusters was variable, prevailing those containing only 1 (16 clusters, ∼52%), 2 (8 clusters, ∼26%), and 3 MRPs (3 clusters, 10%). Clusters 1, 2 and 16 (numbered according their average reactivity, see below) showed the highest *n* values, with 23, 7 and 12 sequences, respectively (Fig 2B). Further sequence alignments allowed us to refine 7 antigenic motifs from clusters with *n ≥* 3 sequences (the consensus sequences are indicated in Fig 2C). Great differences in reactivity were observed when comparing among clusters, with clusters 1 and 2 showing significantly higher average reactivity (Fig 2D). Clusters 7 to 31, on the other hand, yielded consistently low reactivity values, slightly above the established cutoff (Fig 2D). Intra-cluster differences in reactivity values were also observed, particularly for cluster 1 (Fig 2D). As shown in Fig 2E, even when a significant correlation between the relative reactivity and sequence identity was found, some peptides from cluster 1 presented great dispersion in their antigenicity in spite of the homogeneous identity values within the cluster. Given that sequences within a cluster are structurally (>50% identity) and hence also likely antigenically related, these intra-cluster variations may be attributed to variations in key amino acid positions, though not critical, for the peptide recognition by antibodies with similar specificities.

**Fig 3:**
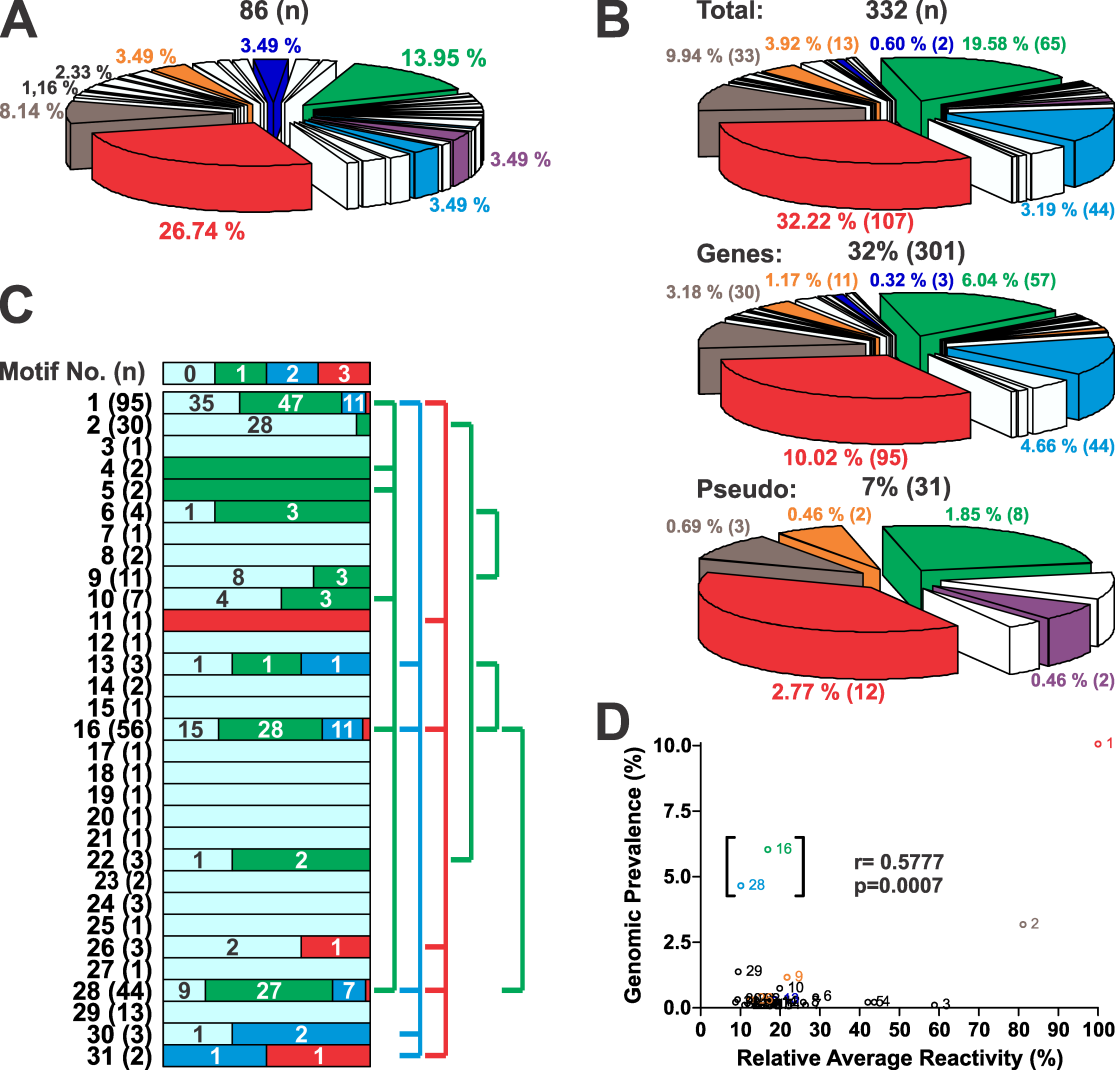
Genomic features of MASP motifs. **A)** Pie chart accounting for the representation (in %) of each cluster to the total number of MASP-derived MRPs identified in the Chagas-chip (*n* = 86). **B)** Pie charts depicting the representation of each cluster in the total augmented list of MASP proteins derived from the *in silico* motif homology-based search (*n* = 332, upper chart), in the CL Brener MASP collection of genes (*n* = 301, middle chart) or pseudogenes (*n* = 31, bottom chart). **C)** The graphic depicts the co-occurrence profile of each motif within a single MASP polypeptide (co-occurrence with 0 (i.e. itself or motif alone), 1, 2 or 3 additional motifs is represented with light blue, green, blue and red boxes, respectively). Total *n* values for each motif are indicated. The number of motif co-occurrence events of each type is indicated in the corresponding box and their % representation is proportional to the box size. Co-occurring motifs are linked by lines at right. **D)** Correlation analysis of the genomic prevalence as a function of the relative average reactivity (in %) of MASPs motifs.

In addition of being the most reactive clusters, clusters 1 and 2 were also amongst the most predominant within the MRP population (26.74 and 8.14% respectively, Fig 3A). To assess the genome prevalence of the identified antigenic motifs, proteome-wide sequence similarity searches were conducted. For clusters with < 3 MRPs, we used the exact sequence(s) as query. As a result of this analysis (performed simultaneously in *T*. *cruzi* strains CL Brener (TcVI), Sylvio X-10 (TcI) and Dm28c (TcI)) 332 deduced proteins were retrieved (S4 Table), all of them annotated as MASPs. As shown in Fig 3B, they account for 32 and 7% of the total *MASP* gene and pseudogene content, respectively. Notably, and despite unusual cases (for example, the preferential association of cluster 26 with pseudogenes and the exclusive association of cluster 28 with genes), the relative representation of each analyzed cluster in the whole *T*. *cruzi* gene and pseudogene content was overall conserved (Fig 3B). Moreover, this distribution is very similar to that of the Chagas-chip (compare Fig 3A and 3B), hence ruling out a possible bias in our MASP sampling. An analysis of cluster co-occurrence performed over the entire population of MASP retrieved sequences revealed that clusters with high genomic representation such as 1, 16 and 28 tend to co-exist within MASP polypeptides (Fig 3C), hence hinting at their possible physical linkage within a unique ‘recombinational block’. Finally, a positive correlation between the relative average reactivity of each motif and its genomic representation was found (r = 0.577, Fig 3D). This correlation index was highly biased by motifs 16 and 28 (within brackets in Fig 3D), since it was greatly increased (r = 0.772 *vs*. r = 0.577) when they were removed from the dataset.

### MASP reactivity is focused to the mature C-terminal region

**Fig 4:**
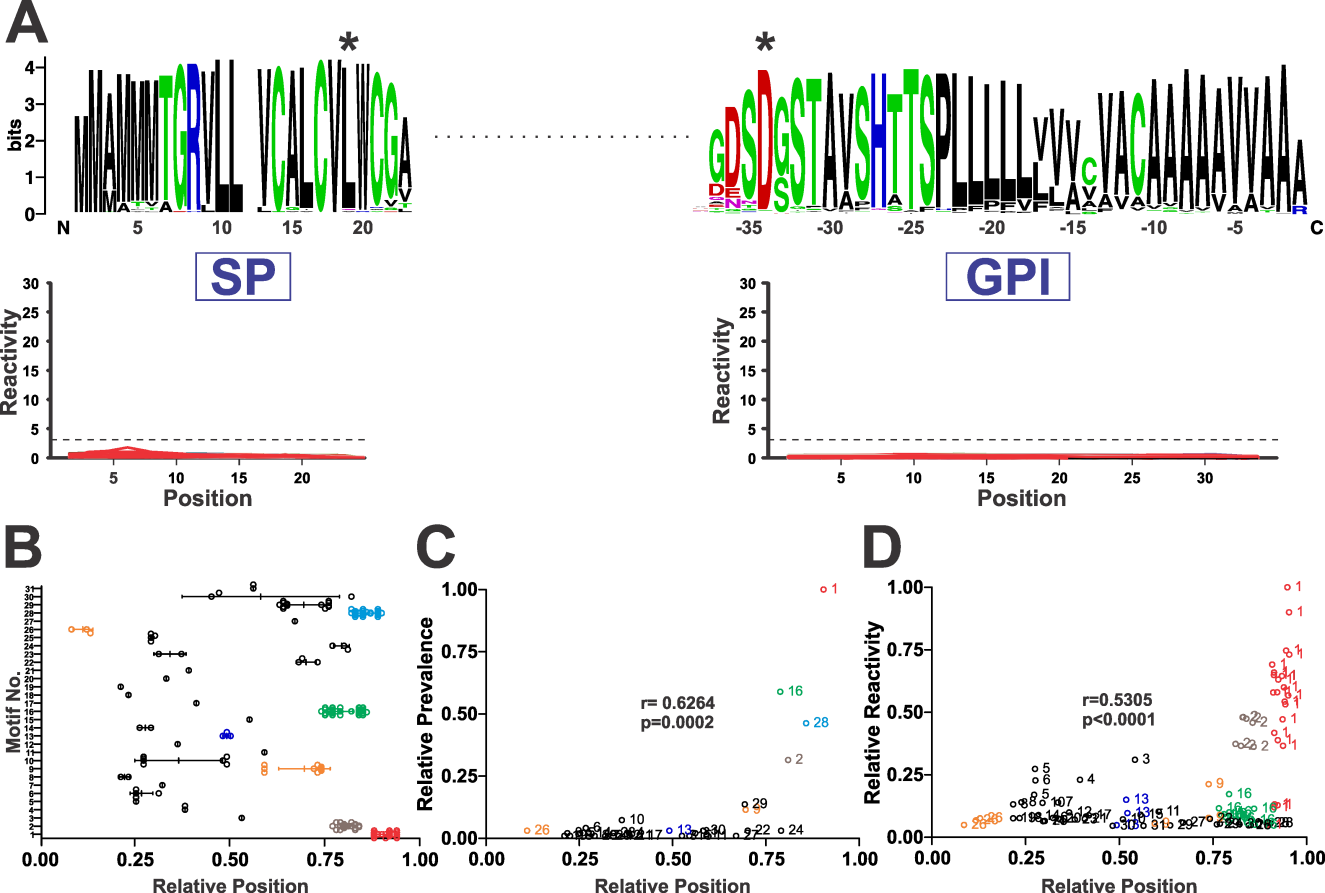
MASP reactivity is focused to the mature C-terminal region. **A)** Lack of reactivity against MASPs SP and GPI-anchoring sequences. Multiple alignments results of MASPs SP and GPI-anchoring sequences included in the Chagas-chip (n = 232) are depicted as *WebLogo* graphics (above) along with their individual reactivity (below). For SP sequences, the predicted initial Meth is denoted as residue 1 whereas for GPI sequences amino acid positions are indicated with negative numbers and the STOP codon is denoted as residue 0. The negative (cutoff) line is indicated with a dashed line. Consensus residues for SP cleavage and GPI addition are denoted by asterisks. **B)** Relative position (RP) of the most reacting peptides (MRPs) corresponding to each MASP antigenic motif. **C)** Correlation analysis of the relative prevalence of MASP motifs as a function of their RP. **D)** Correlation analysis of the relative average reactivity of MASP motifs as a function of their RP. For every correlation, Pearson’s *r* coefficient and the *p*-value are indicated.

We continued our analysis by mapping the position of antigenic motifs within MASP polypeptides. Notably, and despite previous reports claiming that sorting signals (i.e. GPI-addition sequence and/or SP) of MASPs elicited strong humoral responses in chronic Chagasic patients [24,25], all of the Chagas-chip identified motifs lied within the mature region of MASP molecules (Fig 4A). Within the MASP mature regions, antigenic motifs were broadly distributed (Fig 4B). In spite of this, we did not find high dispersion on the relative position index (r.p. index, defined in ‘Experimental’) as calculated for MRPs belonging to the same cluster (Fig 4B). Even for clusters showing apparent high dispersion in r.p. index such as 10, 29 and 30, this was not due to intrinsic ‘motif motility’ but rather to their alternative location in *MASP* genes bearing evident insertions/deletions.

In absolute terms, antigenic sequences tend to accumulate towards the mature *C*-terminal region of MASPs, and this is driven by the positional distribution of highly represented motifs such as 1, 2, 16 and 28 (Fig 4C). In fact, the most predominant motif 1 constitutes the actual *C*-terminus of the mature MASP molecules in which it is embedded, as it includes an absolutely conserved Asp residue which serves as acceptor for the GPI moiety (see position 16 in motif 1 depicted in Fig 2C). Considering that motifs 1 and 2 were also the most reactive ones (Fig 2D), it was not surprising to find a positive correlation when we plotted the relative reactivity vs the relative position for each MASP motif, (r = 0.530, Fig 4D). Overall, these data indicate that antigenic motifs are restricted to the mature region of MASP molecules, being particularly abundant and antigenic towards their *C*-terminal tips.

### Serological validation of MASP antigenic motifs

To validate the results from the Chagas-chip, an extensive ELISA-based analysis was performed on selected MASP motifs. These were prioritized in accordance to the following criteria: i) Reactivity against positive and negative sera in the Chagas-chip; ii) Chagas-chip prevalence; iii) number of constituting sequences; and iv) representation in the *T*. *cruzi* genome. Seven motifs emerged as the most suitable candidates (S5 Table), and 6 of them (motifs 1, 2, 6, 9, 16 and 24) could be successfully cloned and expressed in bacteria as translational fusions to GST (S3 Table). Peptides derived from these same motifs were synthesized and coupled to a carrier BSA protein (S3 Table). An additional motif representative of non-prioritized motifs (motif 11) was also included in the analysis for comparison purposes, though solely as a BSA-coupled peptide. A strict correlation between synthetic peptide and GST-fusion molecule reactivity data was obtained upon evaluation of these motifs, so we will restrict our analysis to GST-fusion molecules data.

**Fig 5:**
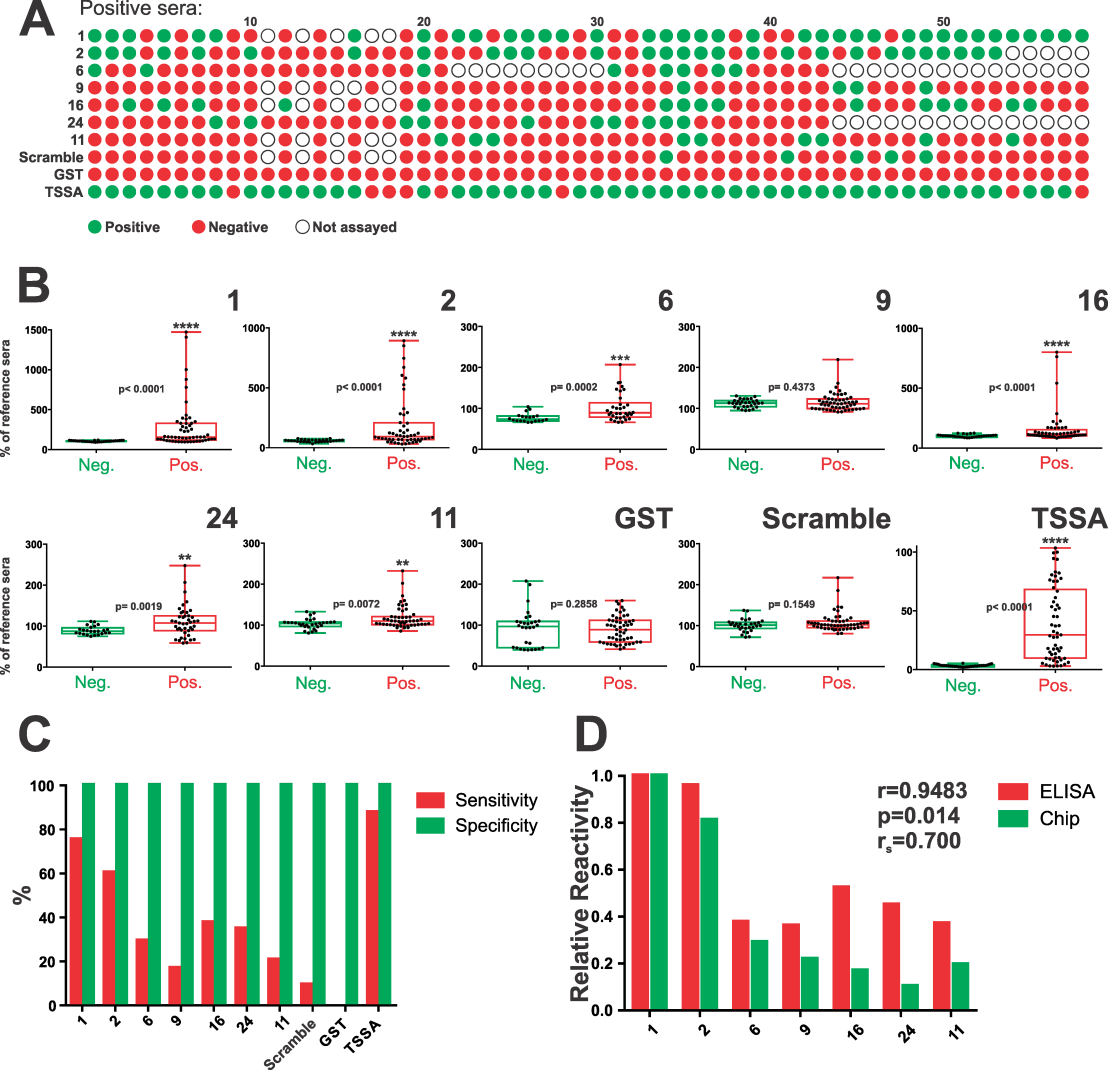
Serological validation of prioritized MASP motifs. **A)** Complete profile of recognition of our panel of positive serum samples (n = 58) towards MASP antigenic motifs. Cutoff-defined true positive and negative results are indicated in green and red, respectively. TSSA (Trypomastigote Small Surface Antigen) was used as positive control whereas a scrambled TSSA peptide and GST (Glutathione *S*-transferase) were used as negative controls. Not evaluated serum/motif combinations are indicated by empty dots. **B)** Box-and-whiskers charts of reactivity values of true positive and negative sera (expressed as % of a reference serum) for each antigenic motif and controls. The level of statistical significance (* and *p*-values) of Mann-Whitney non parametric test are indicated. **C)** Column chart showing % sensitivity (red columns) and specificity (green columns) for each prioritized motif and controls. **D)** Correlation analysis of ELISA (red columns) and Chagas-chip (green columns) reactivity. Pearson´s *r* coefficient (parametric), *p*-value and Spearman’s *r* coefficient (non- parametric) are indicated.

As shown in Fig 5A, most assayed MASP motifs except for motifs 1 and 2 were seldom recognized by a panel of 58 chronic Chagasic sera, which is consistent with their low-to-moderate Chagas-chip prevalence values (Fig 2A). Paired comparisons between positive and negative sera indicated that prioritized MASP motifs display highly variable and dispersed (i.e. not normally distributed) reactivity, with few sera displaying very high signals and most of them exhibiting reactivity closer to the negative population (Fig 5B). Nonetheless, statistically significant differences between positive and negative sera recognition were found for almost all motifs (Fig 5B). In agreement with Chagas-chip data, motifs 1 and 2 emerged as the most antigenic (75 and 60% sensitivity values, Fig 5C) whereas the remaining motifs (except for motif 9) were similar in their overall reactivity profile with respect to the established cutoff (Fig 5B). Non-prioritized motif 11 displayed the less significant differences respect to the control group (Fig 5B), and the lowest sensitivity index (Fig 5C). From a diagnostic standpoint, all motifs displayed 100% specificity, which is in line with our Chagas-chip screening procedures [40]. However, this parameter should be further assessed under more stringent conditions (i.e. using sera from individuals with leishmaniasis). A positive statistically significant correlation between Chagas-chip- and ELISA-based data was found (Pearson’s r = 0.94, p = 0.01, Fig 5D). This was further accompanied by a Spearman (ranked) correlation coefficient of 0.7, indicating also a positive correlation between both data sets in terms of antigenic hierarchy among MASP motifs (Fig 5D).

To better depict the diagnostic performance of each motif, in a manner that is independent of a single arbitrary cut-off value, a Receiver Operating Characteristic (ROC) analysis was performed. Again, motifs 1 and 2 emerged with the best performances, with AUC values of ~0.8 (S2 Fig). The rest of the prioritized motifs (excluding motif 9) showed lower AUC values, ranging from ~0.78 to ~0.71 (S2 Fig). Though not very high, these AUC values were substantially higher than those obtained for GST and the ‘scrambled’ peptide used as negative controls (S2 Fig). Interestingly, they were higher than those assessed for non-prioritized motif 11, hence supporting our prioritization strategy.

### Prioritized antigenic motifs drive MASP recognition by chronic Chagasic sera

**Fig 6:**
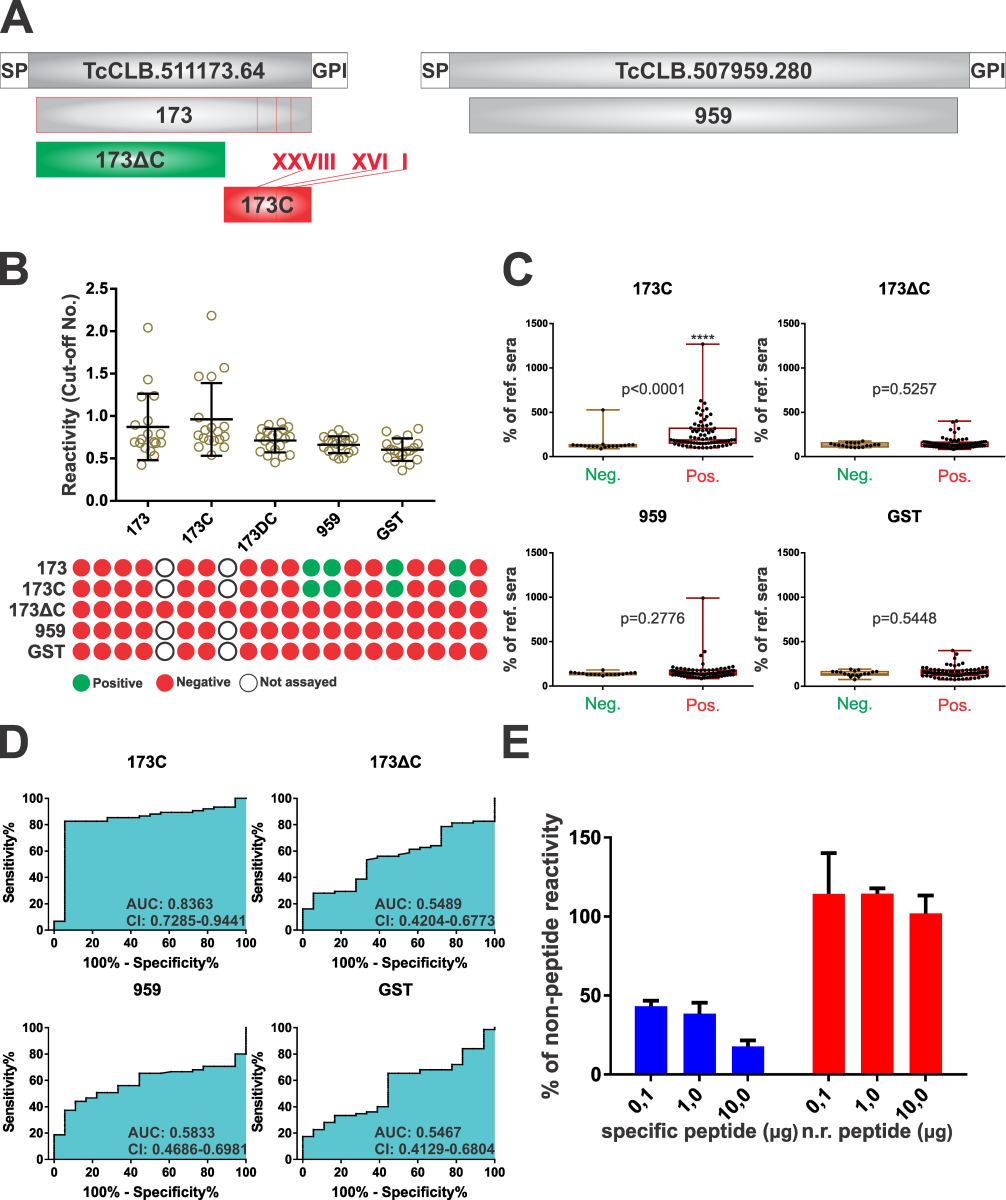
Prioritized antigenic motifs drive MASP reactivity. **A)** Schematic representation of GST- fusion constructs (green) derived upon MASP TcCLB.511173.64 (173, 173C and 173ΔC) or TcCLB.507959.280 (959) generated to perform validation assays. Antigenic motifs (1, 16 and 28) are indicated as red lines within 173 and 173C. **B)** Relative reactivity (left) and one-to-one schematic profiling (right) of Chagasic sera (*n* = 20) against each GST-fusion protein is indicated as in legend to Fig 5. **C)** Box-and-whiskers charts of reactivity values of true positive and negative sera (expressed as % of a reference serum) for the indicated GST-fusion proteins. The level of statistical significance (* and *p*-values) of Mann-Whitney non parametric test are indicated. **D)** ROC curves (%Sensitivity *vs*. 100%- %Specificity) analysis of 173 recombinant constructs and controls. The area under the curve (AUC) and the 95% confidence interval (C.I.) are indicated. **E)** Reactivity values (expressed as the % of reactivity of PBS-added sera) of a Chagasic serum reactive against the GST-fusion protein spanning motif 2 treated with increasing amounts (0.1, 1.0 or 10 μg) of the specific (motif 2 peptide) or non-related (‘scrambled’) peptides.

To assess the impact of prioritized motifs in terms of whole MASP antigenicity, two experimental approaches were undertaken. Firstly, fragments spanning most of the mature regions of MASPs TcCLB.511173.64 and TcCLB.507959.280 (namely 173 and 959, respectively) were cloned and expressed in bacteria as GST-fusions. The former (173) contains 3 of the Chagas-chip-identified motifs (1, 16 and 28), 2 out of which (motifs 1 and 16) were prioritized and included in our serological validation. MASP 959, though similar in size and amino acid composition to MASP 173 does not contain any prioritized motif. Two additional GST-fusion proteins were derived from MASP 173: 173C, spanning solely its *C*-terminal region with the antigenic motifs, and 173ΔC, in which this region has been deleted. Sequences and further details of these recombinant molecules are provided in Fig 6A and S3 Fig.

A preliminary analysis with a reduced number of positive sera revealed that 173 reactivity is driven by its *C*-terminal region, which bear the antigenic motifs. Recognition of 173 showed no differences as compared to that of 173C, with a 100% correlation of positive sera and similar reactivity values (Fig 6B). Construct 173ΔC, on the other hand, showed no reactivity amongst the assayed sera, yielding similar diagnostic performance than 959 (lacking antigenic motifs) and GST used as control (Fig 6B). Upon a more extensive ELISA-based analysis, we found that most of the serum samples that reacted against MASP motifs 1, 16 or 28 also recognized construct 173C (Fig 6C). Indeed, the AUC value of 173C was calculated as ~0.84 (Fig 6D), thus very close to that observed for the most antigenic motif 1 (S2 Fig). Again, 173ΔC and 959 exhibited null diagnostic power (AUC values of ~0.55 and ~0.58, respectively), similarly to GST (Fig 6C and 6D).

To further address this issue, we performed competitive ELISA assays. Motif 2 was chosen because the corresponding GST-fusion molecule yielded well above-the-cutoff reactivity values when assayed against selected Chagasic sera (see Fig 5B). In addition, and due to cloning issues, this construct spanned the motif 2 embedded within > 100 residues of another MASP sequence (TcCLB.503761.40) from the Chagas-chip (see S3 Table). Plates were coated with the GST-fusion molecule and assayed with reactive sera as described above. Before being added to the plate, serum samples were incubated with different amounts of the ‘scrambled’ peptide (negative control) or the peptide spanning motif 2 (S3 Table). As shown in Fig 6E, pre-incubation with motif 2 peptide, but not with the ‘scrambled’ peptide yielded significant and dose-dependent inhibition of GST-motif 2 recognition. Taken together, these results strongly suggest that the reactivity of recombinant MASP polypeptides can be explained by or ascribed to Chagas-chip-identified antigenic motifs.

## Discussion

Peptide arrays constitute a robust, fast and straightforward approach for the discovery of serological biomarkers with potential diagnostic value. This is because they allow the simultaneous discovery of antigens and the exquisite mapping of their linear B-cell epitopes in a highly-parallelized manner [51]. In the particular case of *T*. *cruzi* MASPs, displaying a striking ‘mosaic-like’ structure along their variable central region [3], the application of this technology turned out especially appropriate. As shown here, serological evaluation of high-density, *T*. *cruzi*-derived peptide arrays [40] revealed a quite complex antigenic landscape for this family. Overall, 86 antigenic motifs were identified and mapped to 69 out of 232 MASP molecules sampled. These could be grouped into 31 clusters of structurally- and likely antigenically-related sequences, and representative members of 7 of these clusters were further characterized using conventional methods.

In general terms, our findings indicate that the antigenicity of MASP motifs displays significant positive correlation with i) their genome dosage and ii) their relative position within the corresponding MASP. The former aspect fits nicely with the well-established model of post-transcriptional regulation of gene expression in trypanosomatids [16]. In such scenario, gene dosage adjustments are predicted to correlate with variations in the mRNA and thereby protein content. In the case of MASP antigenic motifs, which are differentially distributed among different members of a large family of genes, a second assumption should be made: a rather similar probability of expression of different ‘alleles’ during the infection. Concurrent expression of multiple MASP variants has been extensively shown both *in vitro* and in infected mouse models [15,17–22]. Moreover, it is worth noting that several positive sera recognized multiple MASP motifs (including combinations of not co-occurring motifs according to our analysis in Fig 3D). Disregarding antibody cross-recognition issues, these findings are consistent with the likely co-expression of several MASPs *in vivo*, during human infections.

Positional and co-occurrence analysis suggested the possibility of linkage groups containing several antigenic motifs, some of which (such as that containing motifs 1, 16 and 28) are particularly prevalent among the overall collection of MASP genes/pseudogenes. Interestingly, the region between these co-occurring motifs was also found to be highly conserved among different MASPs (S4 Fig), further supporting this idea. The existence of such structures, acting as undividable blocks during intra-family recombination events is compatible with the overall ‘mosaic-like’ structure of MASP molecules, made up of different combinations of a large repertoire of short sequence motifs [3,15]. The putative presence of insertion sites for transposons flanking these ‘recombinational blocks’ as well as the overall role of such genetic elements in the generation and upholding of the large MASP repository deserve to be analyzed.

A remarkable feature regarding the position of antigenic motifs determined here is the above mentioned *C*-terminal constraint. Despite being counterintuitive, this antigenic architecture seems to be a rule rather than an exception for *T*. *cruzi* surface antigens [13,52–54]. According to topology predictions, mature *C*-terminal regions of GPI-anchored molecules are apposed to the lipid bilayer, embedded within the dense parasite glycocalix and hence hindered from circulating antibodies. Moreover, mounting evidence indicates that their ‘solubilization’ by endogenous phospholipase(s) is not a very prevalent mechanism in protozoan parasites [14,23,55]. In the case of MASPs, and similarly to mucins [13,56], another possible hindrance towards immune recognition of their mature *C*-terminal domains is the high frequency of Ser/Thr residues located in these regions, prone to undergo glycosylation [15]. To this respect, we performed general *in silico* glycosylation analysis by means of NetOGlyc and NetNGlyc predictive algorithms. As a result of this analysis, we observed that most identified motifs (though not motif 1, which turned out as the most antigenic one) are predicted to be barely glycosylated, and embedded within a hypo-glycosylated molecular context, which would support their antigenic exposure (S4 Fig). For some of them, this hypo-glycosylated environment has been corroborated by a recent glycoproteomic survey [19].

To place the current findings into perspective, we will briefly discuss previous attempts to tackle MASP antigenicity. Firstly, and with the aim of characterizing MASP expression, the group of Dr Bartholomeu raised an antiserum against a MASP-derived peptide (namely peptide7), selected according to an *in silico* MEME-based prioritization analysis [15]. MASPs bearing peptide7 and variations thereof were included in our analysis, although they did not result in an antigenic determinant according to the Chagas-chip. Interestingly, however, peptide7 location is highly conserved in close *C*-terminal proximity to motif 2. In a subsequent work, the same group prioritized 110 peptides based on *in silico* B-cell epitope prediction and used them to evaluate antibody responses in acutely infected mice [18]. Fifteen of the most reactive of these peptides were also included in our array and, again, none of them exhibited significant recognition by human chronic Chagasic sera. This observation suggests that antigen and epitope characterizations in animal models using defined parasite populations serve to reveal either differences in gene expression by the parasite (when infecting different hosts) or in recognition by the immune system of different hosts during different infection phases (or both), but are of little value for discovery of vaccine targets and/or diagnostically relevant markers.

More recently, Serna *et al*. showed that a peptide bearing a quite complex array of MHC I, MHC II and B-cell epitopes, and restricted to a single MASP molecule (TcCLB.511603.380) was recognized by a panel of chronic Chagasic patients [57]. Unfortunately, these results were published *a posteriori* of the Chagas-chip design, and hence this sequence was not included in our assays. However, peptides displaying substantial structural homology to this peptide yielded consistent negative results (S5 Fig). Lastly, the group of Dr Osuna reported strong humoral responses in chronic Chagasic patients against 15mer peptides spanning highly conserved sequences derived from the *N*-terminal SP or the hydrophobic *C*-terminal GPI anchoring signal of MASPs [24,25]. Moreover, higher titers of circulating antibodies towards a MASP GPI-anchoring motif-derived peptide were found among *T*. *cruzi*-infected individuals displaying gastrointestinal symptoms, suggesting that this sequence may function as a novel serological marker of disease-associated pathology [25]. The authors proposed that these responses are triggered and/or sustained by a quantitatively very minor fraction of ‘immature’ MASPs bearing non-cleaved sorting signals that were found inside parasite MVs [24,25]. In strike contrast, our results indicate that antigenic motifs are restricted to the mature region of MASP molecules. As shown in Fig 4A, peptides entirely derived from the SP or GPI-anchoring regions from the 232 tested MASPs, including the exact sequences evaluated in [24,25] yielded consistent negative results in our 8 chip assays using independent human sera pools (see also [40] for raw peptide reactivity data). Although differences in immune responses in different subjects between studies might account for this discrepancy, we propose that our results are accurate for two main reasons. Firstly, Chagas-chip reliability is high as long as it represents an unbiased vast-coverage sample of these consensus sequences. Secondly, the lack of SP and GPI antigenicity better fits with the extensively characterized and efficient mechanisms -both in *Eukarya* and particularly in trypanosomatids- for efficient cleavage and degradation of these sorting signals during processing of GPI-anchored molecules [26,27].

The serological recognition of prioritized MASP motifs seems to be highly variable and dispersed (i.e. not normally distributed) among the *T*. *cruzi* seropositive population, with few sera displaying very high signals and most of them exhibiting reactivity closer to the negative population. This may be attributed in part to the fact that patients in this study were likely infected with distinct *T*. *cruzi* strains, thus displaying high level of MASP polymorphisms among them. Except for motifs 1 and 2, the rest of the MASP motifs were seldom recognized by chronic Chagasic sera. From a serodiagnosis standpoint, and even if motifs 1 and 2 exhibited relatively good AUC values, their overall performances are not good enough for routine implementation in currently available tests, although their potential applicability in other still unmet diagnostic needs should be further evaluated [58,59]. Finally, it is worth noting that statistically significant correlation between Chagas-chip and ELISA-based data was obtained, hence further validating the applicability of the Chagas-chip platform as a powerful tool for high-throughput identification of relevant *T*. *cruzi* antigenic motifs.

## Supporting information

S1 Table Curated MASP list included in the Chagas-chip. (DOCX)

S2 Table Oligonucleotides used in this study. (DOCX)

S3 Table Sequences used to perform serological validation of MASP antigenic motifs. (DOCX)

S4 Table Analysis of genomic representation of cluster-derived MASP motifs. (DOCX)

S5 Table Summary of the most relevant characteristics of the prioritized motifs. (DOCX)

S1 Fig Chagas-Chip output example, antigenic peak definition and overall MASP reactivity calculation. **A)** Chart depicting an example of the Chip-derived output plotted as the Mean Reactivity (average of all positive sera pools) vs. amino acid position (taking as residue 1 the predicted initial Meth residue) for an emerging positive MASP (TcCLB.507071.100). The signal peptide (SP) and glycosylphosphatidyl inositol (GPI)-anchoring predicted sequences are indicated as blue boxes below the *x*-axis. Sequences corresponding to the most reactive peptide (MRP) within each antigenic peak are indicated. The entire antigenic peak bearing the peptide SEREDDEENDEEEDG as MRP is red-shaded. Dashed line indicates the cut-off calculated for the whole chip. The complete amino-acidic sequence of MASP TcCLB.507071.100 is indicated below. SP and GPI predicted sequences are underlined and each MRP is colored according to figure. Red-shaded box represents the sequence of the entire antigenic peak mentioned above. Cumulative Reactivity calculation for TcCLB.507071.100 (as the arithmetic sum of individual positive peaks antigenicity values) is shown above the sequence. **B)** Detailed view of the antigenic peak bearing the peptide SEREDDEENDEEEDG as MRP showed red-shaded in A). The sequence of each overlapping 15 mer peptide contributing to the peak is shown in red.
(TIF)

S2 Fig ROC curves analysis of prioritized MASP motifs and controls. The area under the curve (AUC) and the 95% confidence interval (C.I.) are indicated.
(TIF)

S3 Fig Recombinant constructs used for the validation of MASP motifs contribution to whole polypeptide antigenicity. *ClustalW* alignment between recombinant fragments 173, 173ΔC, 173C and 959 and the parental MASPs full-length protein sequences (TcCLB.511173.64 and TcCLB.507959.280). SP and GPI sequences are underlined. Antigenic peptides within each sequence are shown as colored boxes (1: red, 16: light blue, 28: dark blue).
(TIF)

S4 Fig Glycosylation analysis of prioritized MASP motifs. **A)** Chart depicting NetNGlyc and NetOGlyc glycosylation predictions superimposed to the relative positions of antigenic motifs (shaded color boxes). SP and GPI consensus sequences coverage is shown as blue empty boxes. **B)** Glycosylation predictions over WebLogo graphics of MASPs regions bearing antigenic motifs 1, 2 and 16. GPI sequence (upper panel) is boxed and omega site is indicated in all three panels (asterisk).
(TIF)

S5 Fig Antigenic features of MASP peptides related to a MASP antigenic motif. Sequence alignment between the MASP peptide proposed as vaccine candidate by Serna et al. [54], and most related peptides evaluated in the Chagas-chip. The % identity and the mean reactivity signal for each peptide is indicated.
(TIF)

## References

[1] Stuart K, Brun R, Croft S, Fairlamb A, Gurtler RE, et al (2008) Kinetoplastids: related protozoan pathogens, different diseases. J Clin Invest 118: 1301–1310. doi: 10.1172/JCI33945 18382742 10.1172/JCI33945 PMC2276762

[2] Buscaglia CA, Kissinger JC, Aguero F (2015) Neglected Tropical Diseases in the Post-Genomic Era. Trends Genet 31: 539–555. doi: 10.1016/j.tig.2015.06.002 26450337 10.1016/j.tig.2015.06.002

[3] El-Sayed NM, Myler PJ, Bartholomeu DC, Nilsson D, Aggarwal G, et al (2005) The genome sequence of Trypanosoma cruzi, etiologic agent of Chagas disease. Science 309: 409–415. doi: 10.1126/science.1112631 16020725 10.1126/science.1112631

[4] Berriman M, Ghedin E, Hertz-Fowler C, Blandin G, Renauld H, et al (2005) The genome of the African trypanosome Trypanosoma brucei. Science 309: 416–422. doi: 10.1126/science.1112642 16020726 10.1126/science.1112642

[5] Ivens AC, Peacock CS, Worthey EA, Murphy L, Aggarwal G, et al (2005) The genome of the kinetoplastid parasite, Leishmania major. Science 309: 436–442. doi: 10.1126/science.1112680 16020728 10.1126/science.1112680 PMC1470643

[6] Campetella O, Sanchez D, Cazzulo JJ, Frasch AC (1992) A superfamily of Trypanosoma cruzi surface antigens. Parasitol Today 8: 378–381. 15463546 10.1016/0169-4758(92)90175-2

[7] Freitas LM, dos Santos SL, Rodrigues-Luiz GF, Mendes TA, Rodrigues TS, et al (2011) Genomic analyses, gene expression and antigenic profile of the trans-sialidase superfamily of Trypanosoma cruzi reveal an undetected level of complexity. PLoS ONE 6: e25914 doi: 10.1371/journal.pone.0025914 22039427 10.1371/journal.pone.0025914 PMC3198458

[8] Acosta-Serrano A, Almeida IC, Freitas-Junior LH, Yoshida N, Schenkman S (2001) The mucin-like glycoprotein super-family of Trypanosoma cruzi: structure and biological roles. Mol Biochem Parasitol 114: 143–150. 11378194 10.1016/s0166-6851(01)00245-6

[9] Buscaglia CA, Campo VA, Frasch AC, Di Noia JM (2006) Trypanosoma cruzi surface mucins: host-dependent coat diversity. Nat Rev Microbiol 4: 229–236. doi: 10.1038/nrmicro1351 16489349 10.1038/nrmicro1351

[10] Kawashita SY, da Silva CV, Mortara RA, Burleigh BA, Briones MR (2009) Homology, paralogy and function of DGF-1, a highly dispersed Trypanosoma cruzi specific gene family and its implications for information entropy of its encoded proteins. Mol Biochem Parasitol 165: 19–31. doi: 10.1016/j.molbiopara.2008.12.010 19393159 10.1016/j.molbiopara.2008.12.010

[11] Cuevas IC, Cazzulo JJ, Sanchez DO (2003) gp63 homologues in Trypanosoma cruzi: surface antigens with metalloprotease activity and a possible role in host cell infection. Infect Immun 71: 5739–5749. doi: 10.1128/IAI.71.10.5739-5749.2003 14500495 10.1128/IAI.71.10.5739-5749.2003 PMC201075

[12] Llewellyn MS, Messenger LA, Luquetti AO, Garcia L, Torrico F, et al (2015) Deep sequencing of the Trypanosoma cruzi GP63 surface proteases reveals diversity and diversifying selection among chronic and congenital Chagas disease patients. PLoS Negl Trop Dis 9: e0003458 doi: 10.1371/journal.pntd.0003458 25849488 10.1371/journal.pntd.0003458 PMC4388557

[13] Buscaglia CA, Campo VA, Di Noia JM, Torrecilhas AC, De Marchi CR, et al (2004) The surface coat of the mammal-dwelling infective trypomastigote stage of Trypanosoma cruzi is formed by highly diverse immunogenic mucins. J Biol Chem 279: 15860–15869. doi: 10.1074/jbc.M314051200 14749325 10.1074/jbc.M314051200

[14] Lantos AB, Carlevaro G, Araoz B, Ruiz Diaz P, Camara Mde L, et al (2016) Sialic Acid Glycobiology Unveils Trypanosoma cruzi Trypomastigote Membrane Physiology. PLoS Pathog 12: e1005559 doi: 10.1371/journal.ppat.1005559 27058585 10.1371/journal.ppat.1005559 PMC4825991

[15] Bartholomeu DC, Cerqueira GC, Leao AC, daRocha WD, Pais FS, et al (2009) Genomic organization and expression profile of the mucin-associated surface protein (masp) family of the human pathogen Trypanosoma cruzi. Nucleic Acids Res 37: 3407–3417. doi: 10.1093/nar/gkp172 19336417 10.1093/nar/gkp172 PMC2691823

[16] Clayton CE (2014) Networks of gene expression regulation in Trypanosoma brucei. Mol Biochem Parasitol 195: 96–106. doi: 10.1016/j.molbiopara.2014.06.005 24995711 10.1016/j.molbiopara.2014.06.005

[17] Atwood JA 3rd, Weatherly DB, Minning TA, Bundy B, Cavola C, et al (2005) The Trypanosoma cruzi proteome. Science 309: 473–476. doi: 10.1126/science.1110289 16020736 10.1126/science.1110289

[18] dos Santos SL, Freitas LM, Lobo FP, Rodrigues-Luiz GF, Mendes TA, et al (2012) The MASP family of Trypanosoma cruzi: changes in gene expression and antigenic profile during the acute phase of experimental infection. PLoS Negl Trop Dis 6: e1779 doi: 10.1371/journal.pntd.0001779 22905275 10.1371/journal.pntd.0001779 PMC3419193

[19] Alves MJ, Kawahara R, Viner R, Colli W, Mattos EC, et al (2017) Comprehensive glycoprofiling of the epimastigote and trypomastigote stages of Trypanosoma cruzi. J Proteomics 151: 182–192. doi: 10.1016/j.jprot.2016.05.034 27318177 10.1016/j.jprot.2016.05.034

[20] Brunoro GV, Caminha MA, Ferreira AT, Leprevost Fda V, Carvalho PC, et al (2015) Reevaluating the Trypanosoma cruzi proteomic map: The shotgun description of bloodstream trypomastigotes. J Proteomics 115: 58–65. doi: 10.1016/j.jprot.2014.12.003 25534883 10.1016/j.jprot.2014.12.003

[21] Nakayasu ES, Sobreira TJ, Torres R Jr., Ganiko L, Oliveira PS, et al (2012) Improved proteomic approach for the discovery of potential vaccine targets in Trypanosoma cruzi. J Proteome Res 11: 237–246. doi: 10.1021/pr200806s 22115061 10.1021/pr200806s PMC3253764

[22] Seco-Hidalgo V, De Pablos LM, Osuna A (2015) Transcriptional and phenotypical heterogeneity of Trypanosoma cruzi cell populations. Open Biol 5(12):150190 doi: 10.1098/rsob.150190 26674416 10.1098/rsob.150190 PMC4703061

[23] Bayer-Santos E, Aguilar-Bonavides C, Rodrigues SP, Cordero EM, Marques AF, et al (2013) Proteomic analysis of Trypanosoma cruzi secretome: characterization of two populations of extracellular vesicles and soluble proteins. J Proteome Res 12: 883–897. doi: 10.1021/pr300947g 23214914 10.1021/pr300947g PMC13615563

[24] De Pablos LM, Diaz Lozano IM, Jercic MI, Quinzada M, Gimenez MJ, et al (2016) The C-terminal region of Trypanosoma cruzi MASPs is antigenic and secreted via exovesicles. Sci Rep 6: 27293 doi: 10.1038/srep27293 27270330 10.1038/srep27293 PMC4897614

[25] Diaz Lozano IM, De Pablos LM, Longhi SA, Zago MP, Schijman AG, et al (2017) Immune complexes in chronic Chagas disease patients are formed by exovesicles from Trypanosoma cruzi carrying the conserved MASP N-terminal region. Sci Rep 7: 44451 doi: 10.1038/srep44451 28294160 10.1038/srep44451 PMC5353755

[26] McConville MJ, Mullin KA, Ilgoutz SC, Teasdale RD (2002) Secretory pathway of trypanosomatid parasites. Microbiol Mol Biol Rev 66: 122–154; table of contents. doi: 10.1128/MMBR.66.1.122-154.2002 11875130 10.1128/MMBR.66.1.122-154.2002 PMC120783

[27] Canepa GE, Mesias AC, Yu H, Chen X, Buscaglia CA (2012) Structural Features Affecting Trafficking, Processing, and Secretion of Trypanosoma cruzi Mucins. J Biol Chem 287: 26365–26376. doi: 10.1074/jbc.M112.354696 22707724 10.1074/jbc.M112.354696 PMC3406720

[28] Atwood JA 3rd, Minning T, Ludolf F, Nuccio A, Weatherly DB, et al (2006) Glycoproteomics of Trypanosoma cruzi trypomastigotes using subcellular fractionation, lectin affinity, and stable isotope labeling. J Proteome Res 5: 3376–3384. doi: 10.1021/pr060364b 17137339 10.1021/pr060364b

[29] Bringaud F, Ghedin E, El-Sayed NM, Papadopoulou B (2008) Role of transposable elements in trypanosomatids. Microbes Infect 10: 575–581. doi: 10.1016/j.micinf.2008.02.009 18467144 10.1016/j.micinf.2008.02.009

[30] Weatherly DB, Peng D, Tarleton RL (2016) Recombination-driven generation of the largest pathogen repository of antigen variants in the protozoan Trypanosoma cruzi. BMC Genomics 17: 729 doi: 10.1186/s12864-016-3037-z 27619017 10.1186/s12864-016-3037-z PMC5020489

[31] Hall JP, Wang H, Barry JD (2013) Mosaic VSGs and the scale of Trypanosoma brucei antigenic variation. PLoS Pathog 9: e1003502 doi: 10.1371/journal.ppat.1003502 23853603 10.1371/journal.ppat.1003502 PMC3708902

[32] Bull PC, Buckee CO, Kyes S, Kortok MM, Thathy V, et al (2008) Plasmodium falciparum antigenic variation. Mapping mosaic var gene sequences onto a network of shared, highly polymorphic sequence blocks. Mol Microbiol 68: 1519–1534. doi: 10.1111/j.1365-2958.2008.06248.x 18433451 10.1111/j.1365-2958.2008.06248.x PMC2440560

[33] Maeland JA, Afset JE, Lyng RV, Radtke A (2015) Survey of immunological features of the alpha-like proteins of Streptococcus agalactiae. Clin Vaccine Immunol 22: 153–159. doi: 10.1128/CVI.00643-14 25540270 10.1128/CVI.00643-14 PMC4308872

[34] Alizon S, Luciani F, Regoes RR (2011) Epidemiological and clinical consequences of within-host evolution. Trends Microbiol 19: 24–32. doi: 10.1016/j.tim.2010.09.005 21055948 10.1016/j.tim.2010.09.005

[35] Queiroz RM, Ricart CA, Machado MO, Bastos IM, de Santana JM, et al (2016) Insight into the Exoproteome of the Tissue-Derived Trypomastigote form of Trypanosoma cruzi. Front Chem 4: 42 doi: 10.3389/fchem.2016.00042 27872839 10.3389/fchem.2016.00042 PMC5097913

[36] Pereira-Chioccola VL, Acosta-Serrano A, Correia de Almeida I, Ferguson MA, Souto-Padron T, et al (2000) Mucin-like molecules form a negatively charged coat that protects Trypanosoma cruzi trypomastigotes from killing by human anti-alpha-galactosyl antibodies. J Cell Sci 113 (Pt 7): 1299–1307. 10704380 10.1242/jcs.113.7.1299

[37] De Pablos LM, Gonzalez GG, Solano Parada J, Seco Hidalgo V, Diaz Lozano IM, et al (2011) Differential expression and characterization of a member of the mucin-associated surface protein family secreted by Trypanosoma cruzi. Infect Immun 79: 3993–4001. doi: 10.1128/IAI.05329-11 21788387 10.1128/IAI.05329-11 PMC3187265

[38] Ohyama K, Huy NT, Yoshimi H, Kishikawa N, Nishizawa JE, et al (2016) Proteomic profile of circulating immune complexes in chronic Chagas disease. Parasite Immunol 38: 609–617. doi: 10.1111/pim.12341 27223052 10.1111/pim.12341

[39] Trocoli Torrecilhas AC, Tonelli RR, Pavanelli WR, da Silva JS, Schumacher RI, et al (2009) Trypanosoma cruzi: parasite shed vesicles increase heart parasitism and generate an intense inflammatory response. Microbes Infect 11: 29–39. doi: 10.1016/j.micinf.2008.10.003 19028594 10.1016/j.micinf.2008.10.003

[40] Carmona SJ, Nielsen M, Schafer-Nielsen C, Mucci J, Altcheh J, et al (2015) Towards High-throughput Immunomics for Infectious Diseases: Use of Next-generation Peptide Microarrays for Rapid Discovery and Mapping of Antigenic Determinants. Mol Cell Proteomics 14: 1871–1884. doi: 10.1074/mcp.M114.045906 25922409 10.1074/mcp.M114.045906 PMC4587317

[41] Aslett M, Aurrecoechea C, Berriman M, Brestelli J, Brunk BP, et al (2010) TriTrypDB: a functional genomic resource for the Trypanosomatidae. Nucleic Acids Res 38: D457–462. doi: 10.1093/nar/gkp851 19843604 10.1093/nar/gkp851 PMC2808979

[42] Dereeper A, Guignon V, Blanc G, Audic S, Buffet S, et al (2008) Phylogeny.fr: robust phylogenetic analysis for the non-specialist. Nucleic Acids Res 36: W465–469. doi: 10.1093/nar/gkn180 18424797 10.1093/nar/gkn180 PMC2447785

[43] Schneider TD, Stephens RM (1990) Sequence logos: a new way to display consensus sequences. Nucleic Acids Res 18: 6097–6100. 2172928 10.1093/nar/18.20.6097 PMC332411

[44] Crooks GE, Hon G, Chandonia JM, Brenner SE (2004) WebLogo: a sequence logo generator. Genome Res 14: 1188–1190. doi: 10.1101/gr.849004 15173120 10.1101/gr.849004 PMC419797

[45] Campo V, Di Noia JM, Buscaglia CA, Aguero F, Sanchez DO, et al (2004) Differential accumulation of mutations localized in particular domains of the mucin genes expressed in the vertebrate host stage of Trypanosoma cruzi. Mol Biochem Parasitol 133: 81–91. 14668015 10.1016/j.molbiopara.2003.09.006

[46] Campo VA, Buscaglia CA, Di Noia JM, Frasch AC (2006) Immunocharacterization of the mucin-type proteins from the intracellular stage of Trypanosoma cruzi. Microbes Infect 8: 401–409. doi: 10.1016/j.micinf.2005.07.008 16253534 10.1016/j.micinf.2005.07.008

[47] Balouz V, Camara Mde L, Canepa GE, Carmona SJ, Volcovich R, et al (2015) Mapping Antigenic Motifs in the Trypomastigote Small Surface Antigen from Trypanosoma cruzi. Clin Vaccine Immunol 22: 304–312. doi: 10.1128/CVI.00684-14 25589551 10.1128/CVI.00684-14 PMC4340888

[48] De Marchi CR, Di Noia JM, Frasch AC, Amato Neto V, Almeida IC, et al (2011) Evaluation of a recombinant Trypanosoma cruzi mucin-like antigen for serodiagnosis of Chagas' disease. Clin Vaccine Immunol 18: 1850–1855. doi: 10.1128/CVI.05289-11 21880857 10.1128/CVI.05289-11 PMC3209031

[49] Canepa GE, Degese MS, Budu A, Garcia CR, Buscaglia CA (2012) Involvement of TSSA (trypomastigote small surface antigen) in Trypanosoma cruzi invasion of mammalian cells. Biochem J 444: 211–218. doi: 10.1042/BJ20120074 22428617 10.1042/BJ20120074

[50] Di Noia JM, Buscaglia CA, De Marchi CR, Almeida IC, Frasch AC (2002) A Trypanosoma cruzi small surface molecule provides the first immunological evidence that Chagas' disease is due to a single parasite lineage. J Exp Med 195: 401–413. doi: 10.1084/jem.20011433 11854354 10.1084/jem.20011433 PMC2193624

[51] Andresen H, Bier FF (2009) Peptide microarrays for serum antibody diagnostics. Methods Mol Biol 509: 123–134. doi: 10.1007/978-1-59745-372-1_8 19212718 10.1007/978-1-59745-372-1_8

[52] Cazzulo JJ, Frasch AC (1992) SAPA/trans-sialidase and cruzipain: two antigens from Trypanosoma cruzi contain immunodominant but enzymatically inactive domains. Faseb J 6: 3259–3264. 1426764

[53] Pitcovsky TA, Buscaglia CA, Mucci J, Campetella O (2002) A functional network of intramolecular cross-reacting epitopes delays the elicitation of neutralizing antibodies to Trypanosoma cruzi trans-sialidase. J Infect Dis 186: 397–404. doi: 10.1086/341463 12134236 10.1086/341463

[54] Alvarez P, Leguizamon MS, Buscaglia CA, Pitcovsky TA, Campetella O (2001) Multiple overlapping epitopes in the repetitive unit of the shed acute-phase antigen from Trypanosoma cruzi enhance its immunogenic properties. Infect Immun 69: 7946–7949. doi: 10.1128/IAI.69.12.7946-7949.2001 11705983 10.1128/IAI.69.12.7946-7949.2001 PMC98897

[55] Silverman JM, Chan SK, Robinson DP, Dwyer DM, Nandan D, et al (2008) Proteomic analysis of the secretome of Leishmania donovani. Genome Biol 9: R35 doi: 10.1186/gb-2008-9-2-r35 18282296 10.1186/gb-2008-9-2-r35 PMC2374696

[56] Camara Mde L, Canepa GE, Lantos AB, Balouz V, Yu H, et al (2017) The Trypomastigote Small Surface Antigen (TSSA) regulates Trypanosoma cruzi infectivity and differentiation. PLoS Negl Trop Dis 11(8): e0005856 doi: 10.1371/journal.pntd.0005856 28800609 10.1371/journal.pntd.0005856 PMC5568413

[57] Serna C, Lara JA, Rodrigues SP, Marques AF, Almeida IC, et al (2014) A synthetic peptide from Trypanosoma cruzi mucin-like associated surface protein as candidate for a vaccine against Chagas disease. Vaccine. 10.1016/j.vaccine.2014.04.026 PMC4058865 24793944

[58] Balouz V, Aguero F, Buscaglia CA (2017) Chagas Disease Diagnostic Applications: Present Knowledge and Future Steps. Adv Parasitol 97: 1–45. doi: 10.1016/bs.apar.2016.10.001 28325368 10.1016/bs.apar.2016.10.001 PMC5363286

[59] da Silveira JF, Umezawa ES, Luquetti AO (2001) Chagas disease: recombinant Trypanosoma cruzi antigens for serological diagnosis. Trends Parasitol 17: 286–291. 11378036 10.1016/s1471-4922(01)01897-9

