# Supplementary material for: High-resolution profiling of linear B-cell epitopes from mucin-associated surface proteins (MASPs) of Trypanosoma cruzi during human infections
Source: PLoS Negl Trop Dis. 2017 Sep 29;11(9):e0005986. doi: 10.1371/journal.pntd.0005986 (PMC5636173; doi:10.1371/journal.pntd.0005986)
Supplement: S1 Table — (DOCX) [file pntd.0005986.s001.docx]

**Table S1. Curated MASP list included in the Chagas-chip.**

| **GI^a^** | **Subgroup^b^** | **Reference source^c^** |
| --- | --- | --- |
| TcCLB.511875.9 | S1 | MEMEs Groups [15] |
| TcCLB.506599.50 | S1 |  |
| TcCLB.511595.30 | S1 |  |
| TcCLB.511597.40 | S1 |  |
| TcCLB.511599.90 | S1 |  |
| TcCLB.511599.120 | S1 |  |
| TcCLB.511603.80 | S1 |  |
| TcCLB.511605.20 | S1 |  |
| TcCLB.509977.80 | S1 |  |
| TcCLB.510037.10 | S1 |  |
| TcCLB.510039.30 | S1 |  |
| TcCLB.507959.280 | S1 |  |
| TcCLB.506965.70 | S1 |  |
| TcCLB.506967.70 | S1 |  |
| TcCLB.511609.30 | S1 |  |
| TcCLB.511611.20 | S1 |  |
| TcCLB.511613.10 | S1 |  |
| TcCLB.510961.10 | S1 |  |
| TcCLB.507237.170 | S1 |  |
| TcCLB.507237.220 | S1 |  |
| TcCLB.508151.20 | S1 |  |
| TcCLB.508109.10 | S1 |  |
| TcCLB.508375.10 | S1 |  |
| TcCLB.509925.10 | S1 |  |
| TcCLB.503665.30 | S1 |  |
| TcCLB.506409.30 | S1 |  |
| TcCLB.506409.90 | S1 |  |
| TcCLB.508389.100 | S1 |  |
| TcCLB.510621.20 | S1 |  |
| TcCLB.509657.30 | S1 |  |
| TcCLB.506973.30 | S1 |  |
| TcCLB.507867.10 | S1 |  |
| TcCLB.509699.210 | S1 |  |
| TcCLB.510561.30 | S1 |  |
| TcCLB.504655.9 | S1 |  |
| TcCLB.511255.80 | S1 |  |
| TcCLB.509975.30 | S1 |  |
| TcCLB.509973.70 | S1 |  |
| TcCLB.511173.50 | S1 |  |
| TcCLB.510553.30 | S1 |  |
| TcCLB.510107.30 | S1 |  |
| TcCLB.510269.20 | S1 |  |
| TcCLB.510275.370 | S1 |  |
| TcCLB.508157.20 | S1 |  |
| TcCLB.508163.50 | S1 |  |
| TcCLB.508163.270 | S1 |  |
| TcCLB.510279.60 | S1 |  |
| TcCLB.508165.120 | S1 |  |
| TcCLB.506671.10 | S1 |  |
| TcCLB.503717.20 | S1 |  |
| TcCLB.505025.130 | S1 |  |
| TcCLB.509081.110 | S1 |  |
| TcCLB.503761.40 | S1 |  |
| TcCLB.508165.420 | S1 |  |
| TcCLB.504039.180 | S1 |  |
| TcCLB.506599.420 | S1 |  |
| TcCLB.506599.100 | S1 |  |
| TcCLB.503859.40 | S1 |  |
| TcCLB.506501.240 | S1 |  |
| TcCLB.506499.40 | S1 |  |
| TcCLB.506765.74 | S1 |  |
| TcCLB.503503.40 | S1 |  |
| TcCLB.510373.90 | S1 |  |
| TcCLB.511089.30 | S1 |  |
| TcCLB.510373.60 | S1 |  |
| TcCLB.503977.30 | S1 |  |
| TcCLB.506759.80 | S1 |  |
| TcCLB.506759.40 | S1 |  |
| TcCLB.506613.50 | S1 |  |
| TcCLB.510371.120 | S1 |  |
| TcCLB.510197.20 | S1 |  |
| TcCLB.509755.40 | S1 |  |
| TcCLB.509753.60 | S1 |  |
| TcCLB.508247.130 | S1 |  |
| TcCLB.503771.90 | S1 |  |
| TcCLB.506139.40 | S1 |  |
| TcCLB.504155.230 | S1 |  |
| TcCLB.504155.160 | S1 |  |
| TcCLB.509545.50 | S1 |  |
| TcCLB.511839.20 | S1 |  |
| TcCLB.511843.10 | S1 |  |
| TcCLB.505297.60 | S1 |  |
| TcCLB.510625.190 | S1 |  |
| TcCLB.510629.70 | S1 |  |
| TcCLB.511603.160 | S1 |  |
| TcCLB.508979.60 | S1 |  |
| TcCLB.506995.70 | S1 |  |
| TcCLB.511667.50 | S1 |  |
| TcCLB.503519.30 | S1 |  |
| TcCLB.503849.40 | S1 |  |
| TcCLB.457979.10 | S1 |  |
| TcCLB.510697.40 | S1 |  |
| TcCLB.507071.20 | S1 |  |
| TcCLB.507071.100 | S1 |  |
| TcCLB.507071.180 | S1 |  |
| TcCLB.508433.10 | S1 |  |
| TcCLB.508433.180 | S1 |  |
| TcCLB.503645.40 | S1 |  |
| TcCLB.506335.100 | S1 |  |
| TcCLB.506459.10 | S1 |  |
| TcCLB.503973.150 | S1 |  |
| TcCLB.503973.190 | S1 |  |
| TcCLB.511255.50 | S1 |  |
| TcCLB.511255.169 | S1 |  |
| TcCLB.511255.200 | S1 |  |
| TcCLB.511255.540 | S1 |  |
| TcCLB.510363.220 | S1 |  |
| TcCLB.504081.530 | S1 |  |
| TcCLB.504081.460 | S1 |  |
| TcCLB.504081.350 | S1 |  |
| TcCLB.504081.90 | S1 |  |
| TcCLB.504081.40 | S1 |  |
| TcCLB.506965.20 | S1 |  |
| TcCLB.508221.970 | S1 |  |
| TcCLB.416605.10 | S1 |  |
| TcCLB.503533.40 | S1 |  |
| TcCLB.503585.10 | S1 |  |
| TcCLB.504031.30 | S1 |  |
| TcCLB.506133.54 | S1 |  |
| TcCLB.506309.30 | S1 |  |
| TcCLB.506877.20 | S1 |  |
| TcCLB.506939.50 | S1 |  |
| TcCLB.505949.80 | S1 |  |
| TcCLB.506131.30 | S1 |  |
| TcCLB.506131.84 | S1 |  |
| TcCLB.506245.200 | S1 |  |
| TcCLB.506423.10 | S1 |  |
| TcCLB.506741.90 | S1 |  |
| TcCLB.506879.10 | S1 |  |
| TcCLB.507231.30 | S1 |  |
| TcCLB.507295.20 | S1 |  |
| TcCLB.508745.20 | S1 |  |
| TcCLB.508759.40 | S1 |  |
| TcCLB.508789.150 | S1 |  |
| TcCLB.510463.140 | S1 |  |
| TcCLB.444331.10 | S2 | Trypomastigote vesicles [23] |
| TcCLB.468217.14 | S2 |  |
| TcCLB.503429.30 | S2 |  |
| TcCLB.503783.80 | S2 |  |
| TcCLB.503875.30 | S2 |  |
| TcCLB.503973.280 | S2 |  |
| TcCLB.504239.220 | S2 |  |
| TcCLB.504239.350 | S2 |  |
| TcCLB.505025.10 | S2 |  |
| TcCLB.506101.10 | S2 |  |
| TcCLB.506321.140 | S2 |  |
| TcCLB.506321.20 | S2 |  |
| TcCLB.506453.30 | S2 |  |
| TcCLB.506499.140 | S2 |  |
| TcCLB.506499.210 | S2 |  |
| TcCLB.506589.140 | S2 |  |
| TcCLB.506599.170 | S2 |  |
| TcCLB.506609.70 | S2 |  |
| TcCLB.506763.150 | S2 |  |
| TcCLB.506763.260 | S2 |  |
| TcCLB.506763.280 | S2 |  |
| TcCLB.506769.30 | S2 |  |
| TcCLB.507091.80 | S2 |  |
| TcCLB.507803.10 | S2 |  |
| TcCLB.507957.200 | S2 |  |
| TcCLB.507957.220 | S2 |  |
| TcCLB.507959.170 | S2 |  |
| TcCLB.508013.110 | S2 |  |
| TcCLB.508163.140 | S2 |  |
| TcCLB.508165.170 | S2 |  |
| TcCLB.508165.210 | S2 |  |
| TcCLB.508165.350 | S2 |  |
| TcCLB.508165.410 | S2 |  |
| TcCLB.508221.550 | S2 |  |
| TcCLB.508253.10 | S2 |  |
| TcCLB.508977.50 | S2 |  |
| TcCLB.508999.150 | S2 |  |
| TcCLB.508999.80 | S2 |  |
| TcCLB.509079.20 | S2 |  |
| TcCLB.509081.20 | S2 |  |
| TcCLB.509081.5 | S2 |  |
| TcCLB.509195.70 | S2 |  |
| TcCLB.509631.20 | S2 |  |
| TcCLB.509905.180 | S2 |  |
| TcCLB.510089.10 | S2 |  |
| TcCLB.510105.310 | S2 |  |
| TcCLB.510163.30 | S2 |  |
| TcCLB.510191.10 | S2 |  |
| TcCLB.510205.50 | S2 |  |
| TcCLB.510275.190 | S2 |  |
| TcCLB.510275.250 | S2 |  |
| TcCLB.510279.120 | S2 |  |
| TcCLB.510361.190 | S2 |  |
| TcCLB.510477.100 | S2 |  |
| TcCLB.510621.49 | S2 |  |
| TcCLB.511099.190 | S2 |  |
| TcCLB.511171.90 | S2 |  |
| TcCLB.511173.270 | S2 |  |
| TcCLB.511173.64 | S2 |  |
| TcCLB.511213.15 | S2 |  |
| TcCLB.511221.50 | S2 |  |
| TcCLB.511399.50 | S2 |  |
| TcCLB.511401.100 | S2 |  |
| TcCLB.511593.30 | S2 |  |
| TcCLB.511797.200 | S2 |  |
| TcCLB.511877.20 | S2 |  |
| TcCLB.504587.30 | S2 | Trypomastigote cDNA library [15] |
| TcCLB.506245.270 | S2 |  |
| TcCLB.506321.50 | S2 |  |
| TcCLB.506615.100 | S2 |  |
| TcCLB.506799.130 | S2 |  |
| TcCLB.508125.140 | S2 |  |
| TcCLB.508165.30 | S2 |  |
| TcCLB.508305.50 | S2 |  |
| TcCLB.508541.110 | S2 |  |
| TcCLB.509979.350 | S2 |  |
| TcCLB.510025.260 | S2 |  |
| TcCLB.510279.140 | S2 |  |
| TcCLB.511173.100 | S2 |  |
| TcCLB.511259.220 | S2 |  |
| TcCLB.506973.20 | S2 | Trypomastigote peptide [15] |
| TcCLB.507859.60 | S2 |  |
| TcCLB.508389.130 | S2 |  |
| TcCLB.508389.154 | S2 |  |
| TcCLB.509195.30 | S2 |  |
| TcCLB.510621.60 | S2 |  |
| TcCLB.511787.10 | S2 |  |
| TcCLB.511797.167 | S2 |  |
| TcCLB.504081.420 | S2 | *T. cruzi* proteome [17] |
| TcCLB.506321.200 | S2 |  |
| TcCLB.508871.40 | S2 |  |
| TcCLB.510239.70 | S2 |  |
| TcCLB.511607.40 | S2 |  |
| TcCLB.506757.50 | S2 | Trypomastigote glycoproteome [28] |
| TcCLB.506765.50 | S2 |  |
| TcCLB.510359.380 | S2 |  |
| TcCLB.504239.220 | S2 | MASP 52 [37] |

^a^Genomic identifiers (GI, TritypsDB.org) for the 232 MASP protein genes composing the initial pool. ^b^Subset assignment (S1: ‘MEMEs’ or S2:‘Trypomastigote’) according to the source. ^c^Reference of the source for each subset.
