# Supplementary material for: High-resolution profiling of linear B-cell epitopes from mucin-associated surface proteins (MASPs) of Trypanosoma cruzi during human infections
Source: PLoS Negl Trop Dis. 2017 Sep 29;11(9):e0005986. doi: 10.1371/journal.pntd.0005986 (PMC5636173; doi:10.1371/journal.pntd.0005986)
Supplement: S2 Table — (DOCX) [file pntd.0005986.s002.docx]

**Table S2.** **Oligonucleotides used in this study.**

| **Name** | **Sequence (5’-3’)^a^** | **Purpose** |
| --- | --- | --- |
| PGEX1 | CCGTCGACGAGCTCGCACTCGAGGCAGCGGCCGCGAAGCTTGCAG | Modification of pGEX1λ-T vector |
| PGEX2 | AATTCTGCAAGCTTCGCGGCCGCTGCCTCGAGTGCGAGCTCGTCGACG |  |
| I FOR | TTTTCTCGAGCCTCCGAAGGAAACACCCGTC | Motif 1 cloning |
| I REV | TTGCGGCCGCTGCCGTCACTGTCGCCA |  |
| II FOR | GGGGCTCGAGGGAGTTGGTTCTGCTGGTGGT | Motif 2 cloning |
| II REV | TTGCGGCCGCCGTCACTGTCGCCTGTATTT |  |
| VI FOR | AAAACTCGAGACAACAGGTAACGGAGGGAA | Motif 6 cloning |
| VI REV | TTGCGGCCGCTGCTGCTGTCGCTGTTCTG |  |
| IX FOR | CCCCCTCGAGGAGAACACACTGCCTGGGGAA | Motif 9 cloning |
| IX REV | TTGCGGCCGCCGTCACTGTCGGCAATCTTT |  |
| XVI FOR | CTCGAGAACAATGATCCAGCAGCTGATGGTGCAGAGACACGAGAAGAAGC | Motif 16 cloning |
| XVI REV | GCGGCCGCTTCTTCTCGTGTCTCTGCACCATCAGCTGCTGGATCATTGTTC |  |
| XXIVFOR | AAAACTCGAGGACGCACAGGGAACACAAGAA | Motif 24 cloning |
| XXIVREV | AAGCGGCCGCTGCCGTCACTGTCGCCA |  |
| 173FOR | GGATCCCCAGGAATTCAAGACATTAA | MASP 173 cloning |
| 173REV | GCGGCCGCGGTGCTGCCGtcactgtc |  |
| 173ΔCFOR | GGATCCCCAGGAATTCAAGACATTA | MASP 173ΔC cloning |
| 173ΔCREV | GCGGCCGCATCATTCTCCTCTTCTTTCT |  |
| 173CFOR | GGATCCCCAGgaattcCGGGTGGTGC | MASP 173C cloning |
| 173CREV | gcggccgcggtgctgccgtcactgtc |  |
| 959FOR | ggatccccaggaattcttctgcaataa | MASP 959 cloning |
| 959REV | GcggCCGCGGTGCTGCCGTCCtgTcGC |  |

**^a^**Restriction sites are shown underlined.
