# Supplementary material for: High-resolution profiling of linear B-cell epitopes from mucin-associated surface proteins (MASPs) of Trypanosoma cruzi during human infections
Source: PLoS Negl Trop Dis. 2017 Sep 29;11(9):e0005986. doi: 10.1371/journal.pntd.0005986 (PMC5636173; doi:10.1371/journal.pntd.0005986)
Supplement: S3 Table — (DOCX) [file pntd.0005986.s003.docx]

**Table S3. Sequences used to perform serological validation of MASP antigenic motifs.**

| **Group** | **Peptide** | **Expressed sequence (H_3_N-*GST*…)^a^** |
| --- | --- | --- |
| **1** | LQVAGIKTTTATTGDSC | …GSVDELALEMKTTTATTGDSDGSNQ-COOH |
| **2** | EKQQQSDEAQVQQHQC | …GSVDELALEGVGSAGGHDTGGVSSGSSVPAPGPPSPPAPPEGPSDPPAAPVVDHSAGSSDGKAESSGSNPSNTTGDSSTGDQTSAAAAAHNSSPAEIPAGTTSGTEHTRQEEEEEEEEEDHEKQQQSDEAQFQQHQQHEHPAENGEESAKDKNAIRTNATANTGDSDGGREACRIHRD-COOH |
| **6** | EADDDDDDDDDDGETC | …GSVDELGVCGGLADEETAGSGSGDELPPESQGVETSPQDPQGSQNRAPGGKENITPERIEEADDDDDDDDDDGETKAEEERSTERQSVQEGAAAPDPVSREENLSDSGQEKNQAILSAEDVSLSGSRESNANHTQTEFEEKKDSEKNPPAVEDALTTGNGGNLEAAAAKLAEFIVTD-COOH |
| **9** | PEEDVASREQDGEDTTSEGEC | …GSVDELALEENTLPGEIAEGNLPSPPEEDVASREQDGEDTTSEGEKNVPPPETAATPQSHRDKGSEGTGEDAKATTVTANTTDTKTKIADSDGGRKITSEFAAAKLAEFIVTD-COOH |
| **16** | QTTGDDDPAADGAGTAEGKQC | …GSVDELALENNDPAADGAETREEAAAKLAEFIVTD-COOH |
| **24** | AQSEADADDDDPQRPC | …GSVDELALEDAQGTQETEGTQGTAASPISTSGSSGAQSEADADDDDSQRPNPEGPQNDGTEAGDTHGPSAVSDAAPQAAKAIAAQTNGTVTPGDSDGSGREACRIHRD-COOH |
| **11** | SEREDDEENDEEEDGC |  |

**^a^**MASPs sequences containing the prioritized MASP motifs expressed as C-terminal GST-fusions. The sequences of motifs are underlined.
