# Supplementary material for: High-resolution profiling of linear B-cell epitopes from mucin-associated surface proteins (MASPs) of Trypanosoma cruzi during human infections
Source: PLoS Negl Trop Dis. 2017 Sep 29;11(9):e0005986. doi: 10.1371/journal.pntd.0005986 (PMC5636173; doi:10.1371/journal.pntd.0005986)
Supplement: S4 Table — (DOCX) [file pntd.0005986.s004.docx]

**Table S4. Analysis of genomic representation of cluster-derived MASP motifs.**

| **Cluster^a^** | **Motif^b^** | **Genes (%)^c^** | **Pseudo. (%)^d^** |
| --- | --- | --- | --- |
| **1** | [AT][AVGT][MTIVP]K[TNA][TG][TA][AT][TAM][TS]G[DNE]SD | 95 (10) | 12 (2.7) |
| **2** | E[RK]Q[HQ]QSDE[AT]Q[VF]QQ[HQ] | 30 (3) | 3 (0.7) |
| **3** | VPSLPADSENSKTGC | 1 (0.1) | 0 (0) |
| **4** | MTEKEDGDNEDEEVE | 2 (0.2) | 0 (0) |
| **5** | D[KE]EEDDADGDEEDYD | 2 (0.2) | 0 (0) |
| **6** | [DE][EA]D[ED]DDDDDD[ND]D[NG][DE][AT] | 4 (0.4) | 0 (0) |
| **7** | TENEDDDEDEEENEE | 1 (0.1) | 0 (0) |
| **8** | E[EK]E[ED][DN]DDDDDDEPEE | 2 (0.2) | 0 (0) |
| **9** | V[AD]SRE[QK]DGED[AT]TSE[DG] | 11 (1.2) | 2 (0.5) |
| **10** | [EQ][ID][SA]D[DE][DE]DEGEDEEE | 7 (0.7) | 0 (0) |
| **11** | SEREDDEENDEEEDG | 1 (0.1) | 0 (0) |
| **12** | EGEIDDDSDVQNEIK | 2 (0.2) | 0 (0) |
| **13** | [AEI]E[EQ][EV]EDE[ED][ED]EE[ED][QE]EE | 3 (0.3) | 0 (0) |
| **14** | EE[AE]GSDEEDQDE | 2 (0.2) | 0 (0) |
| **15** | PTLKAPAAPAAPAAA | 1 (0.1) | 0 (0) |
| **16** | [NDK]DPAAD[GAV][AT][EG][AT][RQAP] | 57 (6) | 8 (1.8) |
| **17** | TTIKQPGGIKTPSST | 1 (0.1) | 0 (0) |
| **18** | KKEEEDNDDDDLEEA | 1 (0.1) | 0 (0) |
| **19** | GDDVEDGDGEDEEEG | 1 (0.1) | 0 (0) |
| **20** | EDDDDEEDDSDEAEA | 1 (0.1) | 0 (0) |
| **21** | KEEEEEEDGIEQVEE | 1 (0.1) | 0 (0) |
| **22** | DQTSAAAAANDSSPA | 3 (0.3) | 0 (0) |
| **23** | NGTGENLDGDEEEGD | 2 (0.2) | 0 (0) |
| **24** | AQSEADAD[DV]DDPQR | 3 (0.3) | 0 (0) |
| **25** | TEEAEDEDSDEEEET | 1 (0.1) | 3 (0.7) |
| **26** | ADG[AV]GGGDGSAGE | 3 (0.3) | 2 (0.5) |
| **27** | TEGSQSTLAATDTAN | 1 (0.1) | 1 (0.2) |
| **28** | ENKDANPKETPV[TE]A | 44 (4.7) | 0 (0) |
| **29** | H[SR]HDTDTEDSTKNA | 13 (1.4) | 0 (0) |
| **30** | G[KE][KE]KD[ED]E[QA]E[PE][NE][ER]E | 3 (0.3) | 0 (0) |
| **31** | DEEDKDDDVKDEEEE | 2 (0.2) | 0 (0) |

^a^Clusters of MASP-derived MRPs (1 to 31). ^b^Corresponding motifs derived from the sequence alignment of the elements of each defined cluster used to conduct homology searches in TriTrypDB.org. ^c^Number of genes containing each motif (and its % of representation in the CL Brener *MASP* genome). ^d^Number of pseudogenes containing each motif (and its % of representation in the collection of CL Brener *MASP* pseudogenes).
