# Supplementary material for: High-resolution profiling of linear B-cell epitopes from mucin-associated surface proteins (MASPs) of Trypanosoma cruzi during human infections
Source: PLoS Negl Trop Dis. 2017 Sep 29;11(9):e0005986. doi: 10.1371/journal.pntd.0005986 (PMC5636173; doi:10.1371/journal.pntd.0005986)
Supplement: S5 Table — (DOCX) [file pntd.0005986.s005.docx]

**Table S5. Summary of the most relevant characteristics of the prioritized motifs.**

| **Group^a^** | **Mean Reactivity^b^** | **Max. Reactivity^c^** | **Mean Negative Pools Reactivity^d^** | **Max. Negative Pools Reactivity^e^** | **Prevalence (%,Chagas-Chip)^f^** | **Sequences (Chagas-Chip)^g^** | **Genomic prevalence (CL Brener)^h^** |
| --- | --- | --- | --- | --- | --- | --- | --- |
| **1** | 34.07 | 64.57 | 0.09 | 0.28 | 100 | 22 | 107 |
| **2** | 27.64 | 31.06 | 0.00 | 0.04 | 75 | 7 | 33 |
| **3** | 20.05 | 20.05 | 0.00 | 0.00 | 50 | 1 | 1 |
| **6** | 9.87 | 14.73 | 4.40 | 4.40 | 50 | 2 | 4 |
| **9** | 7.41 | 13.73 | 0.25 | 0.51 | 25 | 3 | 13 |
| **16** | 5.75 | 7.51 | 0.44 | 1.18 | 75 | 12 | 65 |
| **24** | 4.32 | 5.13 | 1.25 | 1.54 | 50 | 2 | 4 |
| **Rest** | 5.96 | 5.91 | n.d. | n.d. | n.d. | 36 | 106 |

**^a^**Prioritized and remaining non-prioritized clusters derived from the Chagas-Chip (Rest). **^b^**Mean value of reactivity against positive (Chagasic) sera for each cluster. **^c^**Maximum value of reactivity against positive sera found within each cluster. **^d^**Mean value of reactivity against negative (non-chagasic, control) sera for each cluster. **^e^**Maximum value of reactivity against negative sera found within each cluster. **^f^**Prevalence (%) against 4 independent positive sera pools confronted towards the Chagas-Chip. **^g^**Number of positive sequences for each cluster present in the Chagas-Chip. **^h^**Number of sequences (genes and pseudogenes) that bear the defining motif of each cluster.
