# Supplementary figures and images for: High-resolution profiling of linear B-cell epitopes from mucin-associated surface proteins (MASPs) of Trypanosoma cruzi during human infections
Source: PLoS Negl Trop Dis. 2017 Sep 29;11(9):e0005986. doi: 10.1371/journal.pntd.0005986 (PMC5636173; doi:10.1371/journal.pntd.0005986)

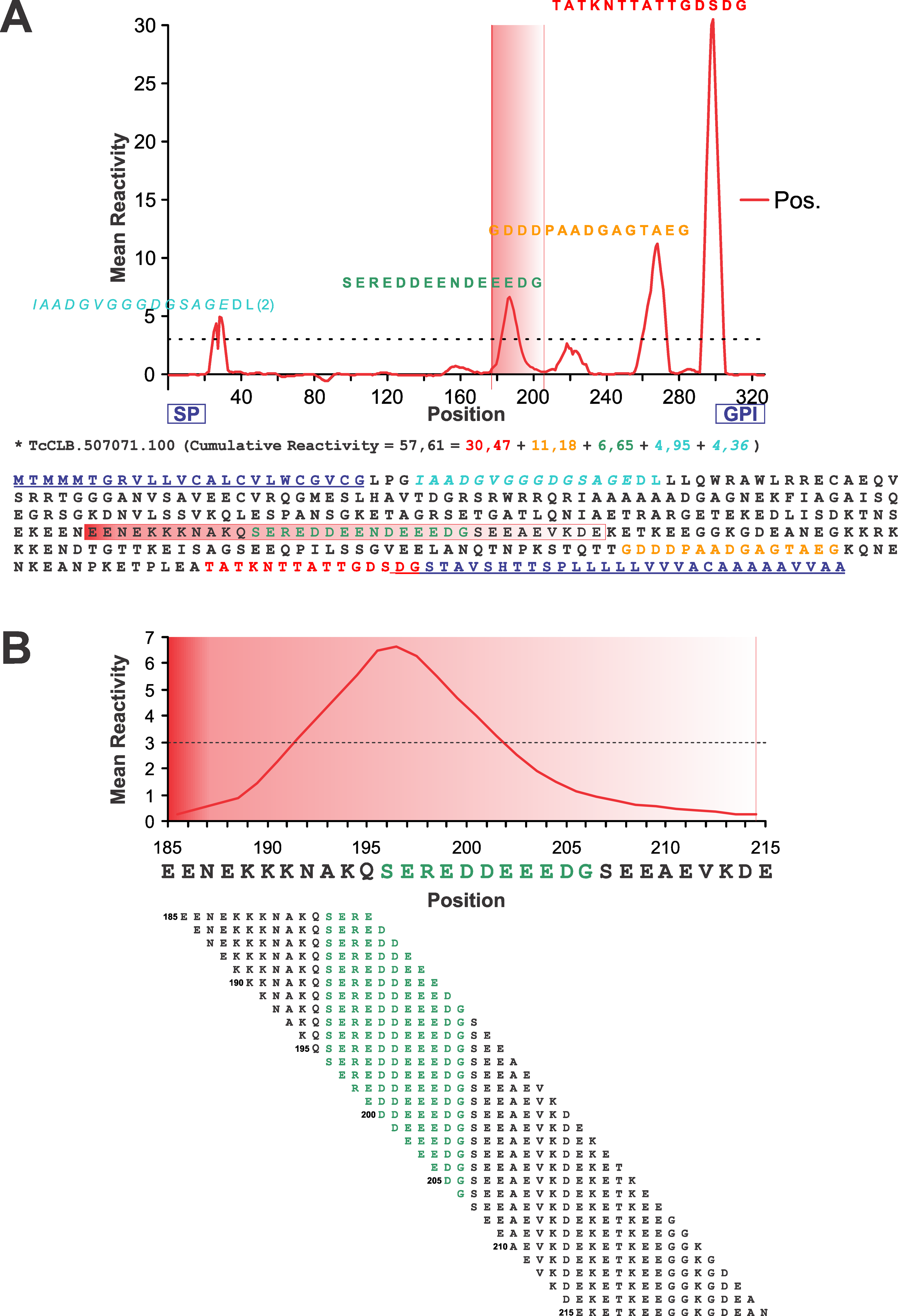

Supplement: S1 Fig — A) Chart depicting an example of the Chip-derived output plotted as the Mean Reactivity (average of all positive sera pools) vs. amino acid position (taking as residue 1 the predicted initial Meth residue) for an emerging positive MASP (TcCLB.507071.100). The signal peptide (SP) and glycosylphosphatidyl inositol (GPI)-anchoring predicted sequences are indicated as blue boxes below the x-axis. Sequences corresponding to the most reactive peptide (MRP) within each antigenic peak are indicated. The entire antigenic peak bearing the peptide SEREDDEENDEEEDG as MRP is red-shaded. Dashed line indicates the cut-off calculated for the whole chip. The complete amino-acidic sequence of MASP TcCLB.507071.100 is indicated below. SP and GPI predicted sequences are underlined and each MRP is colored according to figure. Red-shaded box represents the sequence of the entire antigenic peak mentioned above. Cumulative Reactivity calculation for TcCLB.507071.100 (as the arithmetic sum of individual positive peaks antigenicity values) is shown above the sequence. B) Detailed view of the antigenic peak bearing the peptide SEREDDEENDEEEDG as MRP showed red-shaded in A). The sequence of each overlapping 15 mer peptide contributing to the peak is shown in red. (TIF) [file pntd.0005986.s006.tif]

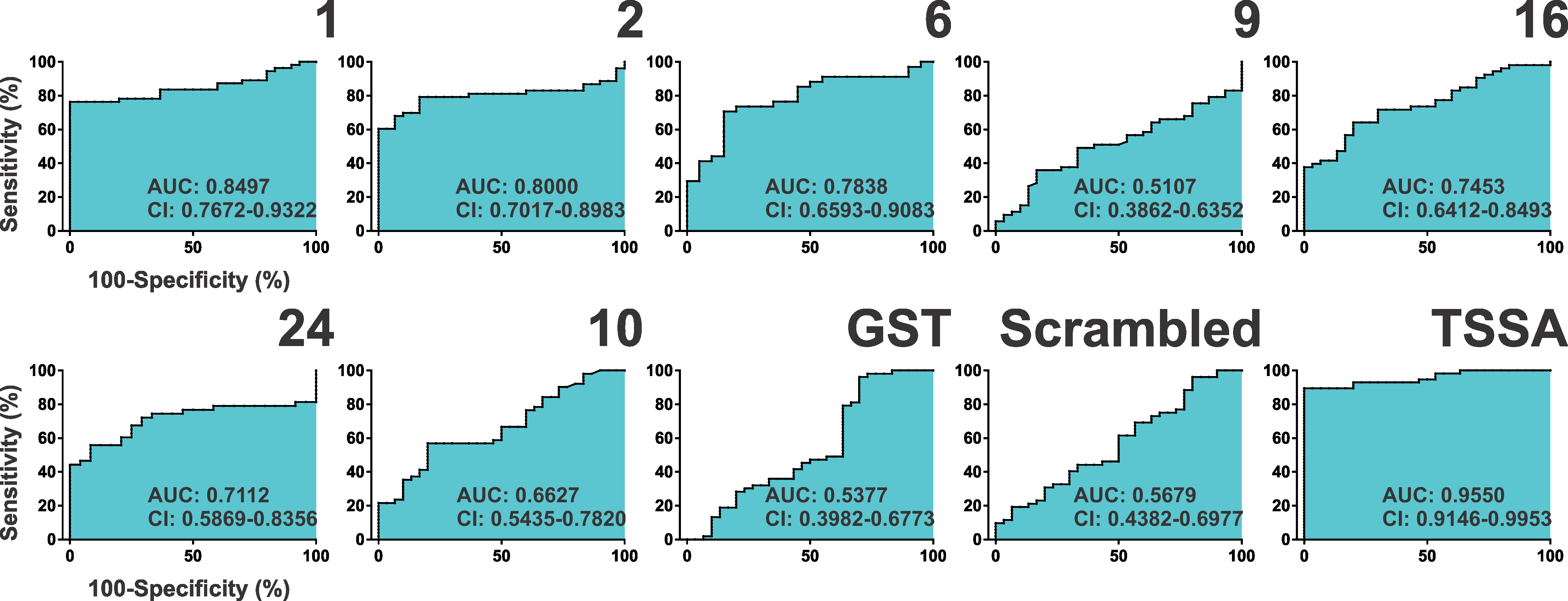

Supplement: S2 Fig — The area under the curve (AUC) and the 95% confidence interval (C.I.) are indicated. (TIF) [file pntd.0005986.s007.tif]

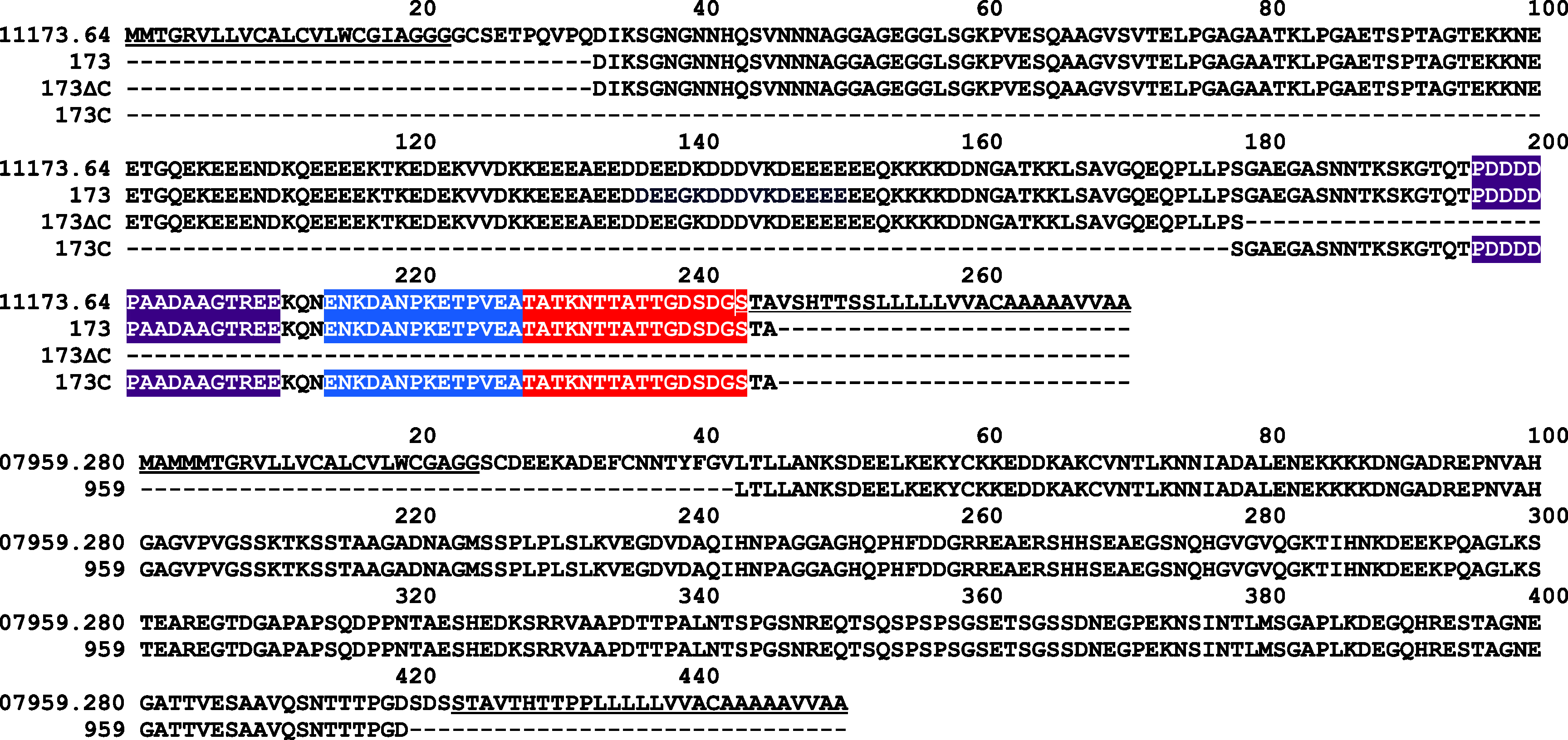

Supplement: S3 Fig — ClustalW alignment between recombinant fragments 173, 173ΔC, 173C and 959 and the parental MASPs full-length protein sequences (TcCLB.511173.64 and TcCLB.507959.280). SP and GPI sequences are underlined. Antigenic peptides within each sequence are shown as colored boxes (1: red, 16: light blue, 28: dark blue). (TIF) [file pntd.0005986.s008.tif]

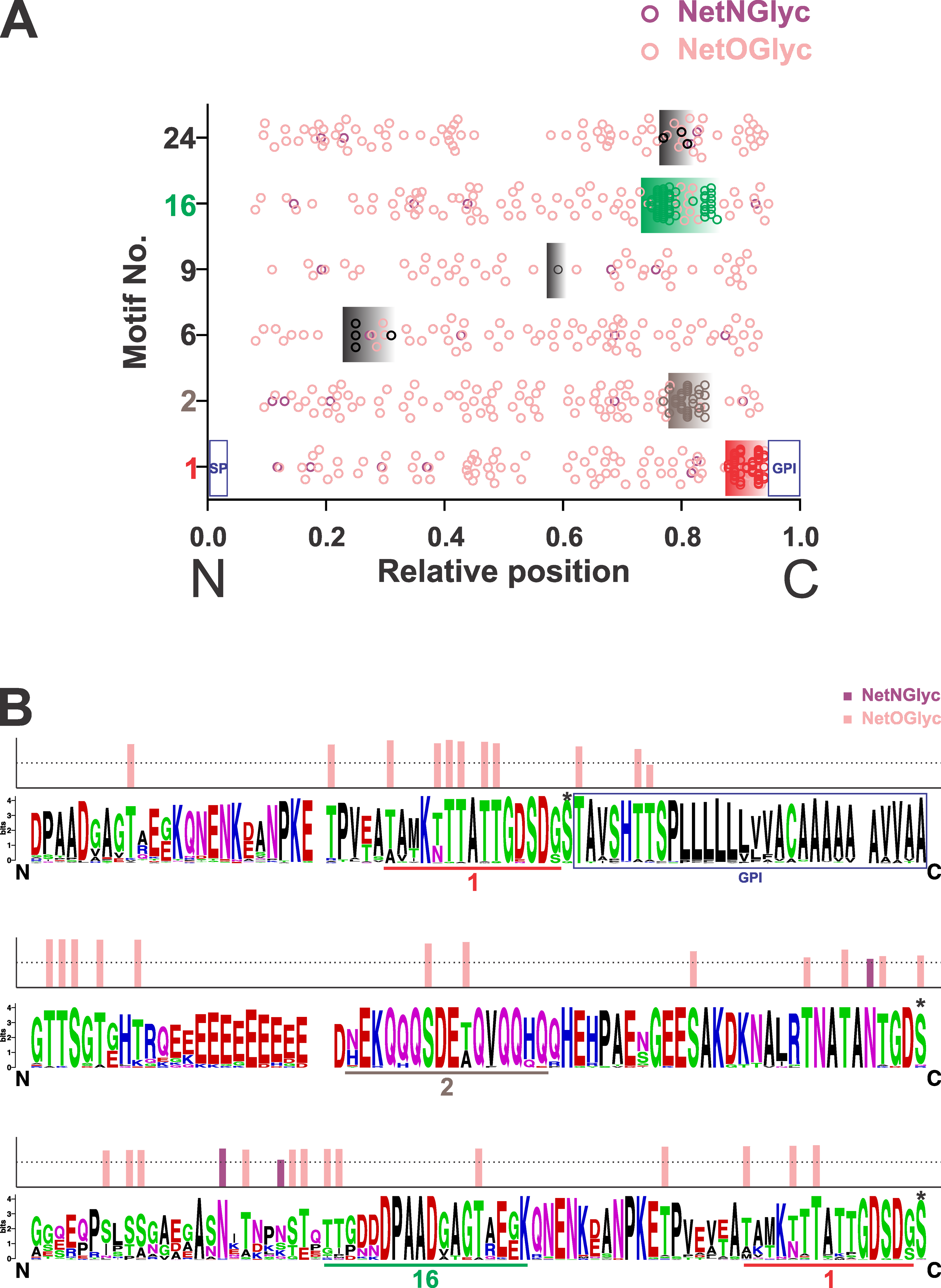

Supplement: S4 Fig — A) Chart depicting NetNGlyc and NetOGlyc glycosylation predictions superimposed to the relative positions of antigenic motifs (shaded color boxes). SP and GPI consensus sequences coverage is shown as blue empty boxes. B) Glycosylation predictions over WebLogo graphics of MASPs regions bearing antigenic motifs 1, 2 and 16. GPI sequence (upper panel) is boxed and omega site is indicated in all three panels (asterisk). (TIF) [file pntd.0005986.s009.tif]

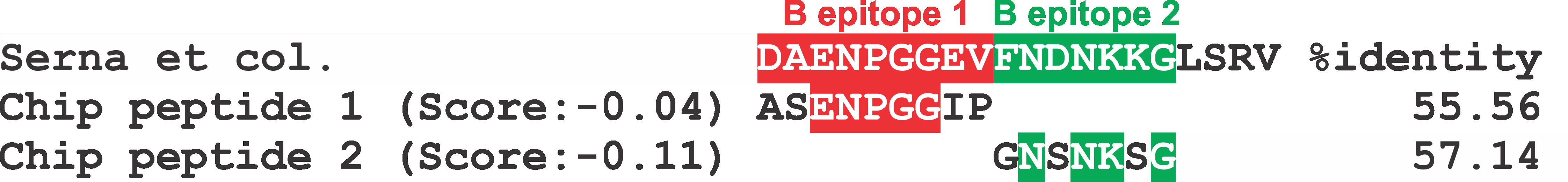

Supplement: S5 Fig — Sequence alignment between the MASP peptide proposed as vaccine candidate by Serna et al. [54], and most related peptides evaluated in the Chagas-chip. The % identity and the mean reactivity signal for each peptide is indicated. (TIF) [file pntd.0005986.s010.tif]
